# Microstructure, Mechanical Response, and Tribological Behavior of Mechanically Alloyed and Microwave-Sintered AA7068/TiB_2_–TiC Hybrid Composites

**DOI:** 10.3390/ma19143072

**Published:** 2026-07-16

**Authors:** Emre Özer

**Affiliations:** Department of Industrial Engineering, Osmaniye Korkut Ata University, Osmaniye 80000, Turkey; mech.eng.emreozer@gmail.com

**Keywords:** 7068 aluminum alloy, hybrid composites, TiB_2_–TiC, powder metallurgy, mechanical properties, wear

## Abstract

In this study, AA7068 aluminum matrix composites reinforced with TiB_2_/TiC were fabricated via mechanical alloying and microwave sintering to investigate the influence of reinforcement content and sintering temperature on microstructure, mechanical properties, and dry sliding wear. Mechanical alloying refined powders, reducing D_50_ from 51.5 µm (AA) to 22.5 µm (AC9) and enhancing dispersion and retention of TiB_2_/TiC particles. XRD confirmed α-Al as the dominant matrix phase, preserved TiB_2_ and TiC phases, and limited MgAl_2_O_4_/ZnAl_2_O_4_ spinel formation. Crystallite refinement and increased lattice microstrain were observed with the addition of reinforcement. Microhardness increased with reinforcement content and sintering temperature, reaching 122.2 HV_0.05_ in AC9-2. At the same time, the highest compressive strength was observed in AC6-2 (431.05 MPa), indicating that optimal load-bearing depends on densification and interfacial integrity rather than hardness alone. AC9-2 exhibited the best wear resistance, with a cumulative specific wear rate of 2.723 × 10^−4^ mm^3^/Nm over 1000 m. SEM-EDS analysis revealed oxide-rich tribolayers, mechanically mixed layers, TiB_2_/TiC fragments, and Fe-rich third-body debris, indicating wear is predominantly hardness-controlled but strongly influenced by microstructural factors. Overall, TiB_2_/TiC hybrid reinforcement improves AA7068 wear resistance through combined hard-particle load-bearing, reduced penetration, tribolayer stability, and third-body effects, offering insight for high-performance hybrid aluminum composites.

## 1. Introduction

7xxx series aluminum alloys have the highest strength potential among commercial Al alloys and are widely used in aerospace, automotive, and other high-performance structural applications [1,2]. AA7068 is a Zn–Mg–Cu-based, precipitation-hardenable alloy with high specific strength and strong potential as a matrix for metal matrix composite design [2,3,4]. When combined with hard ceramic reinforcements, AA7068 can further improve wear resistance and surface-related performance while retaining its intrinsic strength [3,4]. More broadly, Al-based metal matrix composites are considered for aerospace, automotive, defense, transportation, and wear-related applications because reinforcement can enhance specific strength, hardness, thermal stability, and tribological resistance while preserving the low-density advantage of Al alloys [5,6,7].

For high-alloy Al-based composites, powder metallurgy (PM) is advantageous because it is a solid-state route that avoids complete melting and can reduce wettability problems, reinforcement segregation, undesired high-temperature reactions, and inhomogeneous particle distribution [8,9,10]. When combined with high-energy milling, PM promotes compositional homogeneity, reinforcement dispersion, microstructural refinement, and controlled phase development [11,12]. However, the residual porosity, oxidation, and particle clustering at high reinforcement contents remain closely linked to sintering behavior and reinforcement distribution, requiring careful process optimization [13,14,15,16].

Hard ceramic reinforcements are widely used to improve the hardness, strength, and wear resistance of Al-based composites [17,18,19]. TiC is particularly attractive because of its high hardness, wear-resistance contribution, grain-refining ability, and potential for relatively uniform dispersion under suitable processing conditions [17,18,19]. TiB_2_ also provides high hardness, thermal/chemical stability, and resistance to wear-induced degradation [20,21]. Thus, combining the refinement and strengthening effects of TiC with the thermal and tribological stability of TiB_2_ offers a promising hybrid reinforcement strategy for high-strength Al matrix composites.

Although TiB_2_- and TiC-containing hybrid Al matrix systems have been reported [22,23,24,25,26,27,28], most studies focus on specific alloy systems and processing routes. In situ Al–TiB_2_/TiC composites processed by accumulative roll bonding or hot rolling showed improved reinforcement distribution, grain refinement, and mechanical/corrosion behavior [22,23]. Mechanically alloyed and spark-plasma-sintered Al/(TiC+TiB_2_) composites exhibited changes in hardness, elastic modulus, and corrosion behavior [24,25], whereas TiB_2_/TiC/Al laser-alloyed coatings produced compact, wear-resistant layers [26]. In 7xxx alloys, TiB_2_–TiC_x_ or TiC–TiB_2_ additions improved hardness, wear resistance, grain refinement, hot-cracking susceptibility, and weld-joint properties in AA7075 and 7055 systems [27,28]. However, the hybrid effect of TiB_2_ and TiC on phase development, microstructural homogeneity, mechanical response, and tribological behavior in AA7068 remains insufficiently clarified.

Wear studies on AA7068-based alloys and composites show that tribological performance is strongly affected by reinforcement type/content, manufacturing route, and heat treatment. Reinforcements such as graphene nanoplatelets (GNPs), graphite, SiC, MgO, TiO_2_, ZnO, Si_3_N_4_, Al_2_O_3_, ZrO_2_, ZrB_2_, and TiC have been investigated, generally showing improved hardness, load-bearing capacity, and surface stability when particle distribution is sufficiently homogeneous [2,29,30,31,32,33,34,35,36,37,38,39,40]. However, excessive reinforcement may reduce tribological performance through agglomeration, porosity, particle detachment, or embrittlement [2,30]. Although graphene–TiC and high-/medium-entropy alloy (HEA/MEA) hybrid systems have recently been studied in AA7068 [41,42,43,44,45], systematic evaluation of TiB_2_-containing hybrid reinforcement designs, particularly for wear behavior, remains limited.

Mechanical alloying combined with microwave sintering is a promising route for Al matrix composites because it promotes reinforcement dispersion, powder-scale refinement, and rapid densification [46,47,48]. Mechanical alloying improves matrix–reinforcement contact through repeated cold welding and fracture, while microwave sintering can shorten processing time, restrict grain growth, and improve microstructural integrity [46,47]. However, hybrid ceramic-reinforced systems remain process-sensitive because density, hardness, and strength depend on reinforcement content, sintering temperature, and particle distribution; excessive reinforcement may promote porosity, agglomeration, local hotspots, secondary phases, grain coarsening, or interfacial reactions [49,50,51,52,53]. Therefore, in 7xxx-series Al alloys containing two hard ceramic phases, the combined effects of mechanical alloying and microwave sintering on densification, phase stability, microstructure, mechanical properties, and wear mechanisms remain insufficiently understood.

To address this gap, this study fabricated a 7068-type Al–Zn–Mg–Cu-based matrix alloy and TiB_2_–TiC hybrid-reinforced composites by mechanical alloying and microwave sintering. The objective was to determine how TiB_2_/TiC reinforcement and sintering temperature affect densification, phase formation, microstructure, mechanical properties, and dry sliding wear. Pre-mixed and attritor-milled powders were cold-pressed and microwave-sintered at two temperatures, and the resulting structural, mechanical, and tribological responses were correlated with reinforcement composition and processing conditions. The novelty lies in evaluating the complementary TiB_2_/TiC reinforcement potential in AA7068 through a solid-state mechanical alloying–microwave sintering strategy.

## 2. Materials and Methods

### 2.1. Raw Materials and Powder Composition

Al, Zn, Mg, Cu, Fe, Zr, Si, Mn, Ti, and Cr powders were used to produce a 7068-type Al–Zn–Mg–Cu-based matrix alloy with the target chemical composition listed in Table 1, while TiB_2_ and TiC particles served as reinforcement phases. All powders were supplied by Nanografi (Ankara, Türkiye), with alloying powders at above 99.9% purity and reinforcements at above 99.5% purity. The matrix composition was selected within the nominal AA7068 range and near the upper limits of alloying element concentrations to minimize PM-related compositional deviations. The matrix composition is given in Table 1, and the composite compositions and sample codes are listed in Table 2.

### 2.2. Powder Processing and Sintering

Raw powder morphologies were examined using a field-emission scanning electron microscope (SEM; Quanta FEG 650, FEI Company, Hillsboro, OR, USA), and particle size distributions were measured using a laser diffraction particle size analyzer (Mastersizer 3000, Malvern Panalytical Ltd., Worcestershire, UK). Powder batches were prepared according to the target matrix composition and reinforcement volume fractions in Table 1 and Table 2. The required mass of each elemental and reinforcement powder was calculated using their respective true/solid densities listed in Table 3 and weighed on an analytical balance (AS 220.R2, RADWAG Balances and Scales, Radom, Poland). The powders were pre-mixed for 30 min in a 3D mixer (Turbula, Willy A. Bachofen AG, Muttenz, Switzerland) to reduce local compositional gradients, followed by 5 h of mechanical alloying in an attritor mill (MSE Teknoloji, Kocaeli, Türkiye) under argon. This two-step route minimized segregation arising from differences in particle size, morphology, and density by combining Turbula pre-mixing with high-energy attritor milling, which promoted mechanical embedding and redistribution of fine TiB_2_/TiC particles within plastically deformed Al-based powders. Ethanol (7.5 mL) was used as a process control agent. Water recirculation and 10 min milling/10 min rest intervals were applied to limit heating, agglomeration, and sticking. Zirconia balls with a diameter of 10 mm were used at a ball-to-powder ratio of 10:1 and a milling speed of 300 rpm.

After mechanical alloying, the powders were cold compacted at 600 MPa using a hydraulic press (Hürsan, Konya, Türkiye) with petrolatum as a die lubricant. The compacts were heated to 400 °C at 10 °C/min and held for 30 min to remove the lubricant, then microwave-sintered at 550 or 600 °C for 1 h under an argon atmosphere and otherwise identical conditions. Sintering was performed in a microwave sintering system (MicroHeat MH2917, ENERZI Microwave Systems Pvt. Ltd., Belgaum, India) operating at 2540 MHz, with two 1.45 kW magnetrons (2.9 kW total) and a SiC susceptor for hybrid heating. Cylindrical specimens of Ø16 mm × 24 mm were prepared for compression tests, while Ø20 mm × 10 mm specimens were used for other characterizations.

### 2.3. Materials Characterization and Testing

Raw and sintered densities were determined by Archimedes’ principle according to ASTM B962-23 [54]. For microstructural analysis, samples were ground with 600–1200 grit SiC papers, polished with 6 and 1 μm diamond suspensions, and etched for 10 s using Modified Keller solution (1 mL HF, 1.5 mL HCl, 2.5 mL HNO_3_, and 95 mL distilled water). Optical microstructures were observed with an optical microscope (Eclipse LV150N, Nikon Corporation, Tokyo, Japan), and the sintered surfaces were analyzed using a SEM (Sigma 300 VP, Carl Zeiss Microscopy GmbH, Jena, Germany) equipped with energy-dispersive X-ray spectroscopy (EDS).

X-ray diffraction (XRD) analyses were conducted using a diffractometer (Empyrean, Malvern Panalytical B.V., Almelo, The Netherlands) with Cu Kα radiation (λ = 1.5406 Å) at a scan rate of 2°/min. Phase identification was performed using HighScore Plus software (version 5.3a, Malvern Panalytical B.V., Almelo, The Netherlands) [55] and validated using ICSD reference patterns (version 2025.2) [56].

Microhardness was measured according to ASTM B933-25 [57] using a microhardness tester (FM-700, Future-Tech Corp., Kawasaki, Japan) at 50 gf and 15 s; eight measurements were averaged for each sample. Compression tests were performed in accordance with ASTM E9-19(2025)e1 [58] with three replicates, and the compressive yield strength was determined using the 0.2% offset method. Ball-on-disc wear tests were conducted according to ASTM G99-23 [59] using 6 mm 100Cr6 steel balls, a 10 N load, a sliding speed of 0.2 m/s, and a sliding distance of 1000 m. Wear tracks were subsequently examined by SEM–EDS. Wear loss was determined gravimetrically using an analytical balance with a readability of 0.0001 g, and volume loss was calculated by dividing mass loss by the experimentally determined density of each material condition. The specific wear rate was then calculated from volume loss, normal load, and sliding distance. For each material condition, five independent wear specimens were tested at 200, 400, 600, 800, and 1000 m, with one specimen per distance. Since replicate tests at the same sliding distance were not performed, standard deviations were not reported for wear loss and specific wear rate data.

## 3. Results and Discussion

### 3.1. Powder Characterization

**Figure 1:**
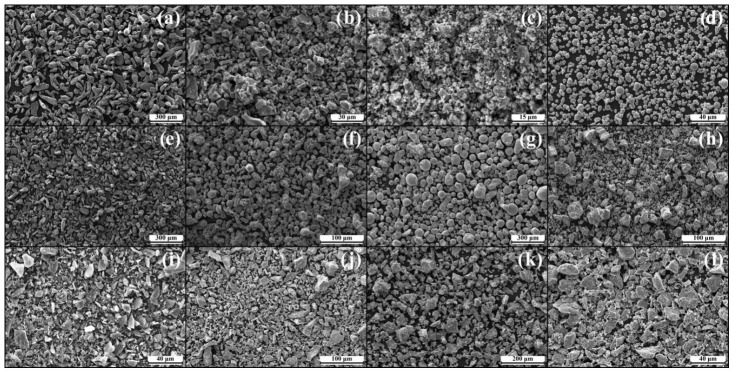
SEM micrographs of the as-received alloy and reinforcement powders showing particle morphology and distribution: (**a**) Al, (**b**) TiB_2_, (**c**) TiC, (**d**) Zn, (**e**) Mg, (**f**) Cu, (**g**) Fe, (**h**) Zr, (**i**) Si, (**j**) Mn, (**k**) Ti, and (**l**) Cr.

Figure 1 shows the initial SEM morphologies of the elemental alloying and TiB_2_/TiC reinforcement powders. Al and Mg powders are irregular, partially elongated, and rough-surfaced, whereas Zn is mainly spherical/near-spherical. Cu and Fe include spherical/hemispherical and irregular particles, while Si, Mn, Ti, Zr, and Cr are mostly angular, fractured, and irregular. These morphological differences can affect packing, interparticle contact, and the deformation–fracture balance during mechanical alloying.

TiB_2_ particles exhibit fine, angular, and irregular morphologies, whereas TiC appears as finer aggregates with local agglomeration. Such agglomeration arises from high specific surface area and surface energy and may promote local stress concentration, porosity, and non-uniform load transfer if dispersion is insufficient [13]. Conversely, repeated cold welding, fracture, and re-welding during mechanical alloying can disperse hard ceramic particles within ductile Al-based powders and increase particle–matrix contact [60]. Thus, Figure 1 provides the baseline for interpreting subsequent densification, microstructural homogeneity, and property evolution.

Table 3 lists the particle size distributions of the as-received alloying and reinforcement powders. The initial mixture contains metallic and ceramic powders with distinct size regimes. Al has a broad and relatively coarse distribution (D_50_ = 52.7 µm; D_90_ = 128 µm), while Mg, Fe, and Ti also show relatively coarse D_50_ values of 44.9, 43.3, and 34.4 µm, respectively. In contrast, Zn, Si, Zr, Cr, and TiC are finer, with D_50_ values of 8.73, 8.61, and 7.74 µm for Zn, Si, and TiC, respectively. TiB_2_ has a D_50_ of 11.8 µm, finer than the main metallic constituents but coarser than TiC.

These particle-size differences govern deformation, fracture, cold welding, and redistribution during mechanical alloying. Coarser ductile Al-based particles undergo flattening, plastic deformation, and repeated fracture while incorporating finer ceramic particles and forming composite powder structures. Uniform dispersion of finer, harder TiB_2_/TiC particles is critical for microstructural homogeneity during compaction and sintering; however, fine TiC particles with small D_10_ and D_50_ values may agglomerate due to their high surface area and energy. Thus, Table 3 indicates that mechanical alloying is essential for homogenization, the dispersion of TiB_2_/TiC, and the formation of a suitable green compact structure. The true/solid density values listed in Table 3 were used to calculate the required powder masses and the theoretical density of each alloy/composite composition.

**Figure 2:**
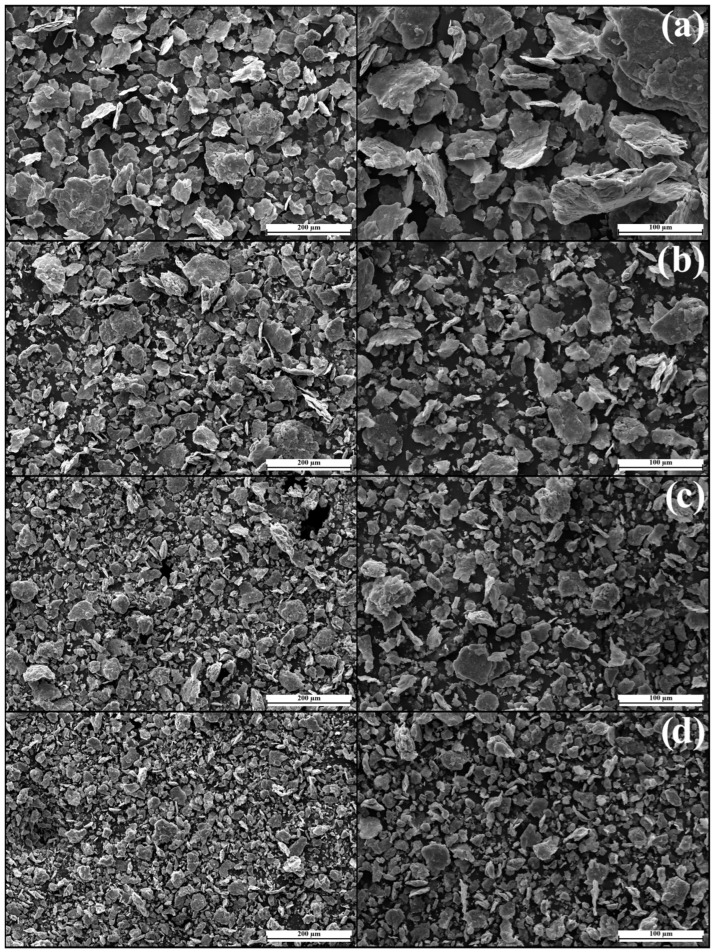
SEM micrographs of the mechanically alloyed powder samples: (**a**) AA, (**b**) AC3, (**c**) AC6, and (**d**) AC9, showing morphologies at 500× and 1000× magnifications.

Figure 2 shows mechanically alloyed AA, AC3, AC6, and AC9 powders. All samples contain irregular, flattened, fractured, and rough-surfaced particles, reflecting repeated cold welding, plastic deformation, fracture, and re-welding. Larger lamellar/flattened and locally cold-welded particles in AA indicate dominant plastic deformation of the ductile Al-based powders. By contrast, AC3, AC6, and AC9 exhibit more fractured, angular, and finer morphologies because hard TiB_2_/TiC particles promote matrix fragmentation and composite powder formation. Similar effects of TiB_2_ particle size, reinforcement content, and milling time on the development of the Al matrix powder and microstructure have been reported [61].

The reinforced samples, especially AC6 and AC9, show a finer and more fractured morphology, indicating that increasing ceramic content shifted the deformation–fracture equilibrium toward fracture. Hard TiB_2_/TiC particles act as local stress concentrators and micro-milling agents, fragmenting the matrix while becoming embedded on Al-based particles or dispersed among them. The coexistence of fractured and flattened particles confirms simultaneous cold welding and fracture toward a dynamic milling equilibrium. Although fine ceramic particles can improve dispersion, insufficient distribution may still cause local agglomeration; therefore, the morphologies in Figure 2 are critical intermediate structures influencing densification, particle–matrix contact, microstructural homogeneity, and final properties.

**Figure 3:**
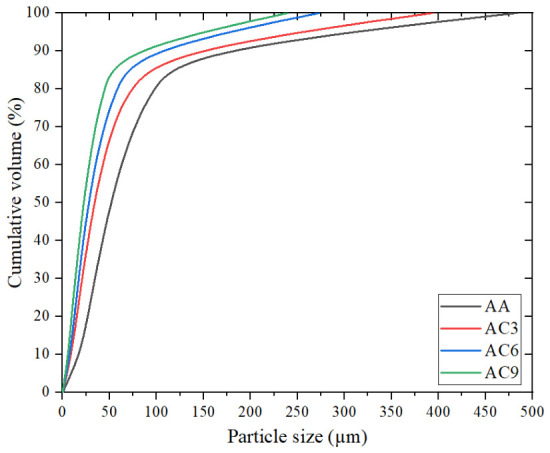
Particle size distribution curves of the mechanically alloyed powders.

Figure 3 shows the particle-size distribution curves of mechanically alloyed AA, AC3, AC6, and AC9 powders, and Table 4 lists the corresponding values. D[3,2] and D[4,3] denote the Sauter and De Brouckere mean diameters, respectively [62]. The reinforced powders shifted toward smaller particle sizes relative to AA, as evidenced by decreases in D_10_, D_50_, and D_90_. These values decreased from 18.6, 51.5, and 130 µm for AA to 9.35, 33.9, and 111 µm for AC3 and to 5.88, 22.5, and 58.8 µm for AC9. This confirms that TiB_2_/TiC particles promoted fragmentation of ductile Al-based powders during mechanical alloying. The pronounced decrease in D_90_ for AC9 should not be interpreted as a simple dilution effect of finer TiB_2_/TiC particles: although AC9 still contains 91 vol.% AA7068 matrix, the matrix powders also underwent severe deformation and fragmentation. The increased TiB_2_/TiC content promoted a fracture-dominated milling regime, reducing the coarse particle fraction and shifting the upper tail of the distribution toward smaller sizes.

Mean diameter values further confirm particle refinement. D[4,3], which is sensitive to coarse particles, decreased from 125 µm for AA to 71, 36.6, and 32.1 µm for AC3, AC6, and AC9, respectively, showing effective breakdown of larger particles. D[3,2] also decreased from 35.7 µm for AA to 14.7 µm for AC9, indicating an increased fine-particle fraction and higher specific surface area. The pronounced leftward shift of AC9 indicates a dominance of fracture over cold welding as reinforcement content increases. Consistent with previous Al–TiB_2_ and Al/(TiC+TiB_2_) studies, reinforcement content, milling conditions, and particle distribution strongly affect powder/microstructure development and properties [24,63].

Across the AC series, TiB_2_/TiC reinforcements acted both as strengthening phases and as fracture-inducing particles during mechanical alloying. The resulting refinement increases interparticle contact prior to sintering and can improve reinforcement distribution, but the higher fraction of fine particles may also promote agglomeration and surface oxidation. Therefore, the consequences of particle refinement in Figure 3 and Table 4 should be interpreted together with densification, porosity, and post-sintering reinforcement distribution.

### 3.2. Microstructure and Phase Analysis

The apparent density ($\rho_{a}$), relative apparent density ($\rho_{ra}$), green density ($\rho_{g}$), relative green density ($\rho_{rg}$), sintered density ($\rho_{s}$), relative sintered density ($\rho_{rs}$), theoretical density ($\rho_{t}$), and sinterability (φ) values of the samples are presented in Table 5. Here, $\rho_{rg}$ and $\rho_{rs}$ denote the relative densities of the green and sintered samples with respect to the $\rho_{t}$, respectively, while φ was calculated using Equation (1) [64]. The $\rho_{t}$ increased from 2.8564 to 3.0246 g/cm^3^ with increasing TiB_2_/TiC content and was calculated by the rule of mixtures using the true/solid densities of the constituent powders listed in Table 3. In Table 5, $\rho_{a}$ refers to the loose powder mixture before compaction, $\rho_{g}$ to the compacted specimen before sintering, $\rho_{s}$ to the Archimedes density after sintering, and $\rho_{t}$ to the rule-of-mixtures value. The $\rho_{ra}$, $\rho_{rg}$, and $\rho_{rs}$ were calculated as the corresponding density divided by the $\rho_{t}$ and multiplied by 100.

(1) $$\begin{array}{c} \varphi =\frac{\rho_{s}-\rho_{g}}{\rho_{t}-\rho_{g}} \end{array}$$

The $\rho_{g}$ increased slightly from 2.6647 g/cm^3^ for AA to 2.7061 g/cm^3^ for AC9; however, the $\rho_{rg}$ decreased from 93.29% to 89.47% with increasing reinforcement content. Thus, although the absolute $\rho_{g}$ rose slightly, TiB_2_/TiC addition reduced compactability relative to $\rho_{t}$. This can be attributed to restricted plastic deformation of ductile Al-based particles, reduced metal–metal contact, and less effective pore closure during compaction. Similar compactability and densification limitations have been reported for ceramic-reinforced Al composites, particularly when reinforcement agglomeration and weak local interparticle contact are present [8,65].

Sintering temperature significantly affected densification. For all samples, $\rho_{s}$ and $\rho_{rs}$ were higher at 600 °C than at 550 °C. For example, the $\rho_{s}$ increased from 2.7997 to 2.8138 g/cm^3^ for AA and from 2.7814 to 2.8273 g/cm^3^ for AC9, while the $\rho_{rs}$ increased from 98.01% to 98.51% and from 91.96% to 93.48%, respectively. This indicates that 600 °C enhanced diffusion, sinter-neck growth, and interparticle bonding. However, $\rho_{rs}$ decreased with increasing reinforcement content because high TiB_2_/TiC fractions reduced sintering efficiency by increasing residual porosity, limiting matrix–matrix contact, and promoting possible particle agglomeration. Therefore, although microwave and field-assisted sintering can enhance densification, the final density remains sensitive to reinforcement distribution, interface quality, processing temperature, and residual porosity [53,66].

The φ values support the same trend. Increasing the sintering temperature improved φ for all compositions, whereas increasing TiB_2_/TiC content limited the extent of densification. The φ values decreased from 0.7042/0.7780 for AA to 0.2364/0.3806 for AC9 at 550/600 °C, respectively. The highest $\rho_{s}$ at 600 °C was obtained in AC6, suggesting a more balanced relationship among reinforcement content, particle distribution, sintering temperature, and densification. In contrast, the lower $\rho_{rs}$ and φ of AC9, despite its higher $\rho_{t}$, indicate stronger agglomeration, poor local interparticle contact, and residual porosity at the highest ceramic content. Thus, TiB_2_/TiC increases $\rho_{t}$, but excessive reinforcement can limit compactability and φ.

**Figure 4:**
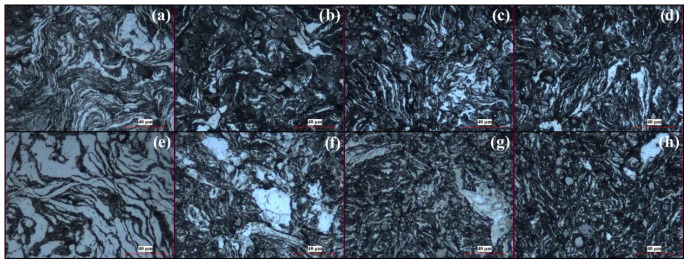
Optical micrographs of the sintered samples showing their microstructures at 500× magnification: (**a**) AA-1, (**b**) AC3-1, (**c**) AC6-1, (**d**) AC9-1, (**e**) AA-2, (**f**) AC3-2, (**g**) AC6-2, and (**h**) AC9-2.

Figure 4 shows optical micrographs of the samples sintered at 550 and 600 °C. The flattened and oriented powder morphology inherited from mechanical alloying remained partially visible after sintering. In AA-1 and AA-2, light Al-based regions and dark boundary/intermediate regions can be associated with lamellar powder structures and interparticle bonding developed during sintering. Since mechanical alloying produces lamellar/oriented morphologies through repeated cold welding, fracture, and re-welding, this initial powder architecture can influence densification and microstructural continuity [60].

In AC3, AC6, and AC9, dark-contrast and fine-particle-rich regions became more pronounced. Although optical microscopy alone cannot definitively identify phases, these contrasts may relate to the distribution of TiB_2_/TiC, oxide/secondary phases, and/or residual porosity. AC3 retained visible-light Al-based regions, whereas AC6 exhibited a finer, more widely distributed dark-contrast morphology, suggesting more effective ceramic dispersion at intermediate reinforcement content. In AC9, denser dark regions and particle-rich areas indicate stronger local agglomeration and/or nonuniform densification. This agrees with the reported difficulty of achieving a homogeneous distribution of reinforcement in ceramic-reinforced Al composites at high reinforcement fractions [13].

Compared with 550 °C, the samples sintered at 600 °C generally showed more pronounced interparticle integration and a denser microstructural appearance, consistent with the higher $\rho_{rs}$ and φ values in Table 5. The higher temperature likely promoted diffusion, sinter-neck growth, and interparticle bonding. However, at high reinforcement contents, ceramic particles still restricted matrix deformation and direct Al–Al contact; therefore, agglomeration and residual porosity were not eliminated. Overall, Figure 4 indicates that TiB_2_/TiC reinforcement contributes to microstructural refinement and particle-rich regions, while local heterogeneity and densification limitations remain important at high reinforcement contents.

**Figure 5:**
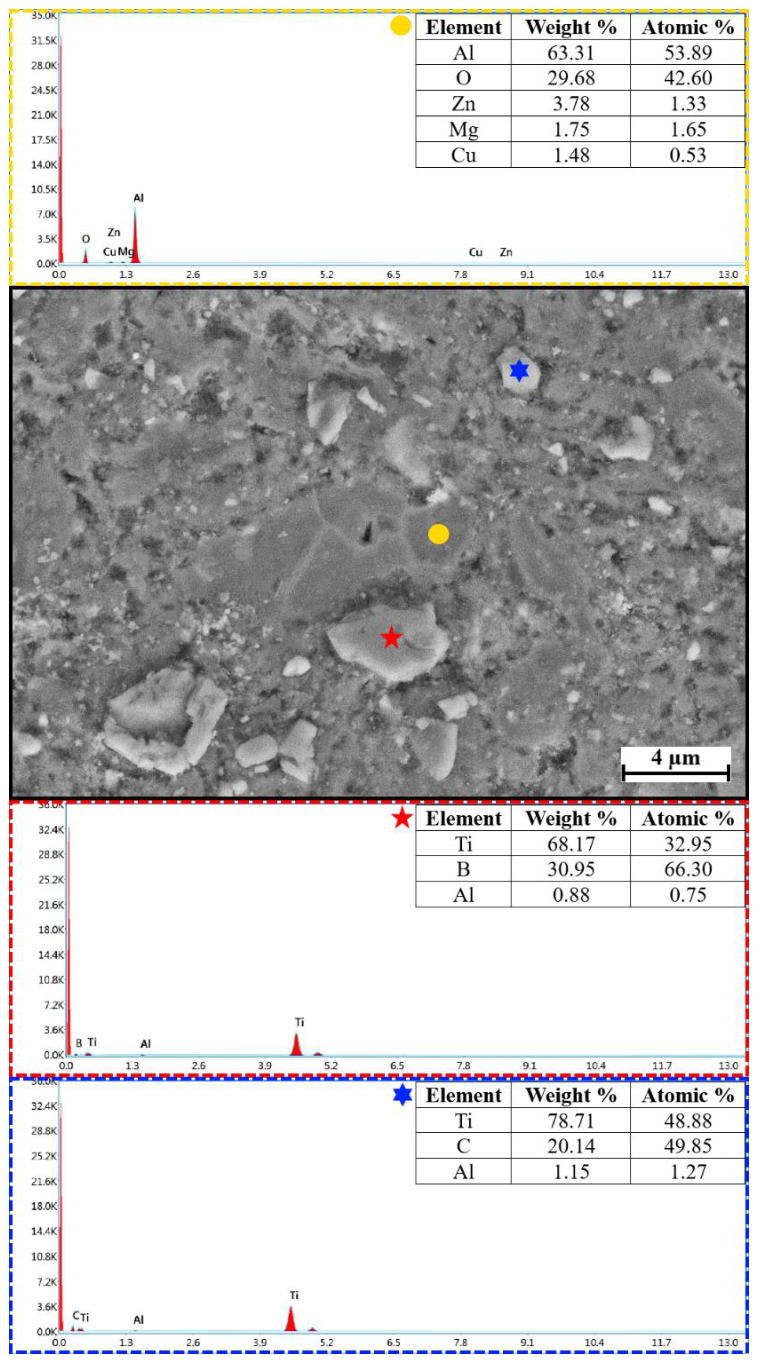
SEM micrograph and corresponding point EDS analyses of the AC9-2 sample. The yellow circle, red star, and blue star mark the EDS analysis locations corresponding to the Al-based matrix region, TiB_2_ particle, and TiC particle, respectively.

Figure 5 shows the SEM image and point EDS analyses of the matrix, TiB_2_, and TiC regions in AC9-2. The matrix region contained mainly Al with detectable Zn, Mg, and Cu, indicating that the 7068-type Al–Zn–Mg–Cu-based composition was largely preserved after sintering. The pronounced O signal may originate from native oxides on Al-based powders, increased surface area after mechanical alloying, and/or local oxygen-containing regions after sintering. In PM Al composites, such oxides can affect interparticle bonding, densification, and interfacial quality [13,66].

The TiB_2_-labeled particle showed dominant Ti and B with very low Al, and the Ti/B ratio was broadly consistent with TiB_2_. The TiC-labeled particle showed dominant Ti and C with an approximately 1:1 atomic ratio. However, because EDS quantification of light elements such as B and C is limited, these point analyses should be considered supportive rather than definitive evidence for phase identification [67]. The SEM image also shows TiB_2_ and TiC particles embedded in or in contact with the Al-based matrix, consistent with mechanical attachment during alloying and the development of particle–matrix contact during sintering.

The retention and distribution of TiB_2_/TiC phases are important for microhardness, compressive strength, and wear behavior because well-dispersed particles with sufficient interfacial contact can strengthen the matrix through load-bearing, impeded dislocation motion, and restricted plastic deformation during wear. However, these effects depend on distribution homogeneity, interface quality, and residual porosity. In Al/(TiC+TiB_2_) composites, the distribution of the reinforcement strongly controls microstructural and mechanical properties [24]. Thus, Figure 5 supports the chemical distinguishability and retention of TiB_2_/TiC reinforcements in AC9-2 after sintering.

**Figure 6:**
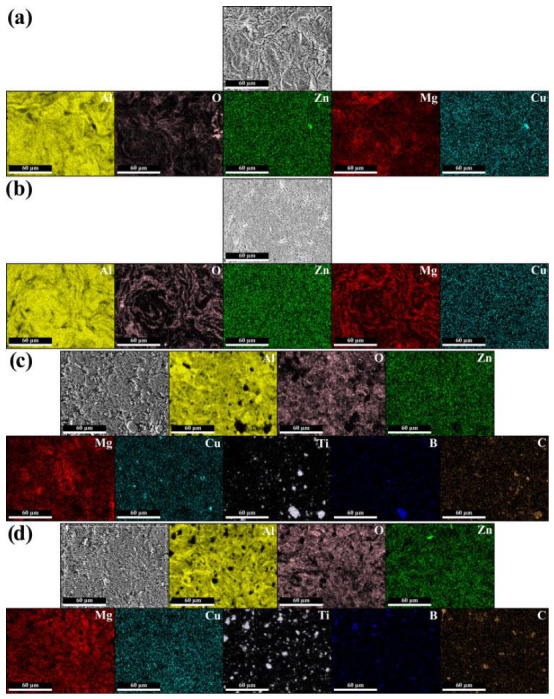
SEM image and corresponding elemental mapping results of the sintered samples: (**a**) AA-1, (**b**) AA-2, (**c**) AC9-1, and (**d**) AC9-2.

Figure 6 presents SEM images and elemental maps of AA-1, AA-2, AC9-1, and AC9-2. In AA-1 and AA-2, Al dominates the analyzed region, while Zn, Mg, and Cu are broadly distributed, indicating that the 7068-type matrix composition was largely retained after mechanical alloying and sintering. Locally intensified O signals may reflect native surface oxides or oxygen-rich regions generated by the increased surface area during milling; such regions can influence interparticle bonding, densification, and mechanical properties.

For AC9-1 and AC9-2, Ti localization in bright particles indicates chemically distinguishable TiB_2_ and/or TiC particles within the matrix. Although B and C signals are detected, their quantitative EDS evaluation remains uncertain due to their low atomic numbers [67]; therefore, mapping should be treated as supportive and interpreted alongside XRD. Nevertheless, Ti, B, and C distributions indicate that TiB_2_/TiC reinforcements were retained as locally identifiable ceramic particles after sintering.

When AC9-1 and AC9-2 are compared, the AC9-2 sample sintered at 600 °C appears to exhibit a more integrated microstructural morphology and a more continuous elemental distribution. This observation is consistent with the higher $\rho_{rs}$ and φ values obtained for AC9 at 600 °C than at 550 °C, as reported in Table 5. However, the presence of localized Ti-rich regions in the AC9 samples indicates that achieving a fully uniform reinforcement distribution at high ceramic contents is challenging and that local particle enrichment may occur. Although repeated cold welding and fracture mechanisms during mechanical alloying promote the embedding and redistribution of ceramic particles within the matrix powders, local clusters may not be eliminated at high reinforcement content [60]. Therefore, the elemental mapping results in Figure 6 indicate that TiB_2_/TiC reinforcements were generally retained and distributed within the 7068-type Al matrix; however, local enrichment of reinforcements and oxygen-containing regions at high reinforcement contents may influence microstructural homogeneity and densification behavior.

**Figure 7:**
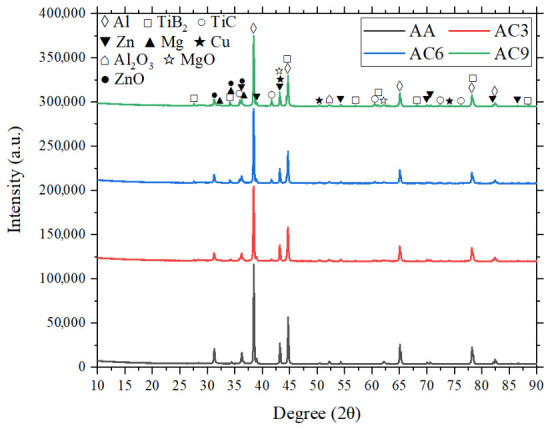
XRD patterns of the mechanically alloyed powder samples.

Figure 7 shows XRD patterns of mechanically alloyed AA, AC3, AC6, and AC9 powders. Phase identification used ICSD 240129 (α-Al), 190893 (TiB_2_), 618925 (TiC), 99783 (α-Al_2_O_3_), 247160 (Zn), 181728 (Mg), 53757 (Cu), 52026 (MgO), and 65121 (ZnO). The dominant peaks correspond to α-Al, confirming the Al-based matrix as the main crystalline phase after mechanical alloying. Low-intensity Zn, Mg, and Cu peaks indicate incomplete solid-solution formation or residual crystalline elemental phases, consistent with the solid-state, kinetically limited nature of mechanical alloying [60].

TiB_2_ and TiC peaks in AC3, AC6, and AC9 indicate that ceramic reinforcements remained as distinct crystalline phases after mechanical alloying. Their peaks are expected to increase with reinforcement content, although overlap with α-Al and alloying-element peaks prevents quantitative assessment based solely on peak intensity. These results confirm the retention of TiB_2_/TiC hybrid reinforcements prior to sintering, a factor known to influence the subsequent microstructure and mechanical properties in Al/(TiC+TiB_2_) systems [24].

Figure 7 also shows weak peaks for α-Al_2_O_3_, MgO, and ZnO. These oxides may originate from native powder-surface oxides and from the higher oxidation susceptibility associated with increased specific surface area during mechanical alloying. In Al-, Mg-, and Zn-containing powder systems, complete oxide removal is difficult; oxygen-rich regions can limit interparticle bonding, the development of sinter necks, and final densification [13]. Overall, α-Al remained dominant, Zn/Mg/Cu were partly retained as detectable crystalline phases, and TiB_2_/TiC reinforcements persisted, while extensive new reaction phases were not evident under the applied milling conditions.

Williamson–Hall (W–H) and Size–Strain Plot (SSP) methods were used to evaluate crystallite size and lattice microstrain from XRD peak broadening using integral breadth values [68,69,70,71]. The W–H approach separates size- and strain-induced broadening according to the uniform deformation model, as given in Equation (2) [71]:

(2) $$\begin{array}{c} \beta_{hkl}\cos\theta =\frac{K\lambda }{D_{WH}}+4\varepsilon \sin\theta \end{array}$$

Here, $\beta_{hkl}$ is the integral breadth, θ is the Bragg angle, K is the shape factor, λ is the X-ray wavelength, D is the crystallite size, and ε is the lattice microstrain. The crystallite size obtained from the W–H method was calculated from the y-intercept of the linear fit using Equation (3):

(3) $$\begin{array}{c} D_{WH}=\frac{K\lambda }{c} \end{array}$$

**Figure 8:**
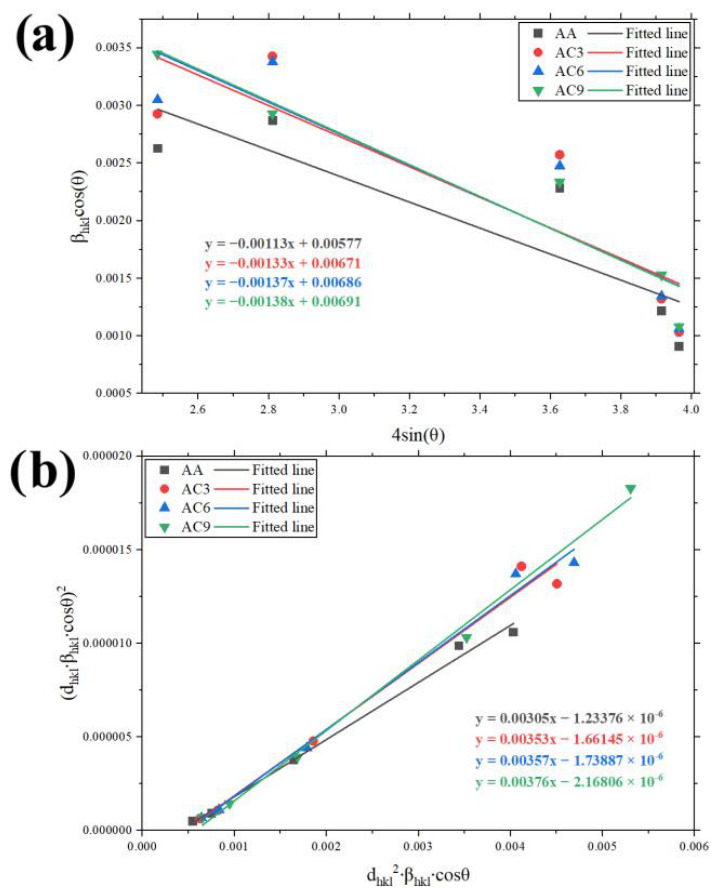
Williamson–Hall (**a**) and Size–Strain Plot (**b**) analyses for crystallite size and lattice microstrain evaluation of the mechanically alloyed samples.

The negative slopes in Figure 8a were not interpreted as physically negative microstrain values; instead, they were treated as fitting-related uncertainty arising from peak overlap, peak selection, instrumental correction, or fitting limitations. Therefore, Table 6 reports microstrain values as comparative absolute magnitudes.

The SSP method was applied as a complementary approach under different peak-profile assumptions, in which size broadening is generally assumed Lorentzian and strain broadening Gaussian [68,69]. The linear SSP relation is given in Equation (4), and crystallite size and microstrain were calculated using Equation (5):

(4) $$\begin{array}{c} {\left(d_{hkl}\;\beta_{hkl}\cos\theta \right)}^{2}=\frac{K\lambda }{D_{SSP}}\left(d_{hkl}^{2}\beta_{hkl}\cos\theta \right)+{\left(\varepsilon /2\right)}^{2} \end{array}$$

(5) $$\begin{array}{c} D_{SSP}=\frac{K\lambda }{m},\varepsilon_{SSP}=2\sqrt{\left|c\right|} \end{array}$$

The interplanar spacing used in the SSP analysis was determined from Bragg’s law, as given in Equation (6) [72]:

(6) $$\begin{array}{c} d_{hkl}=\frac{n\lambda }{2\sin\theta } \end{array}$$

Negative intercepts in Figure 8b were treated as fitting-related limitations rather than physically negative strain values; therefore, the SSP microstrain values in Table 6 are reported as comparative magnitudes. The crystallite sizes obtained from W–H and SSP analyses represent coherently diffracting domains. They should not be directly equated with particle sizes in Table 4 or microstructural features observed by microscopy.

Figure 8 and Table 6 show that TiB_2_/TiC-reinforced powders exhibited smaller crystallite size and higher lattice microstrain than AA. In the W–H analysis, crystallite size decreased from 24.03 nm for AA to 20.07 nm for AC9, while microstrain increased from 0.1130% to 0.1380%. The SSP method showed the same trend, with crystallite size decreasing from 29.51 to 23.94 nm and microstrain increasing from 0.2221% to 0.2945%. Although the absolute values differ because W–H and SSP use different peak-broadening assumptions, the common trend confirms that the addition of TiB_2_/TiC intensified deformation accumulation, lattice distortion, and crystallite-level refinement during mechanical alloying [68,69].

The relatively similar W–H crystallite sizes of AC3, AC6, and AC9 suggest partial saturation of the crystallite refinement after the initial addition of reinforcement. By contrast, the gradual increase in microstrain, especially in SSP, indicates that additional TiB_2_/TiC additions more strongly affected lattice defects and local elastic/plastic deformation than did further subdivision of the crystallites. Thus, Figure 8 and Table 6 show that TiB_2_/TiC reinforcement modifies both particle-scale morphology and crystallite-level defect structure, which may influence sintering response, phase development, and mechanical properties.

**Figure 9:**
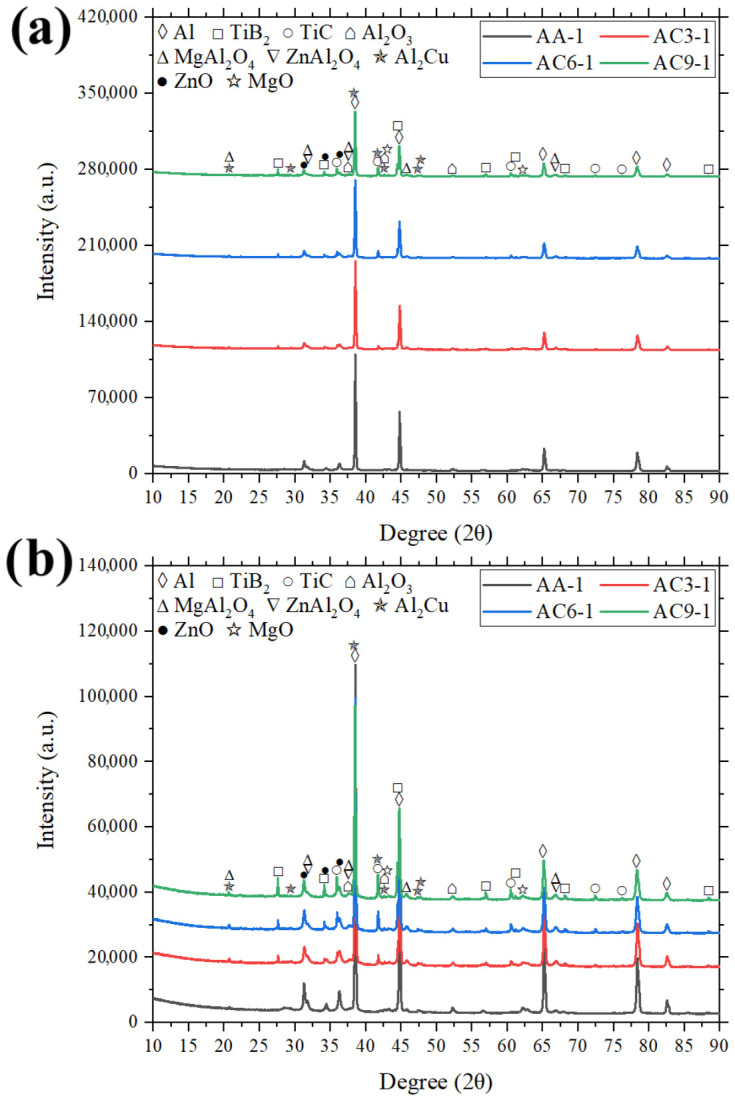
XRD patterns of the samples sintered at 550 °C: (**a**) overall diffraction patterns and (**b**) enlarged low-intensity region for minor peak identification.

**Figure 10:**
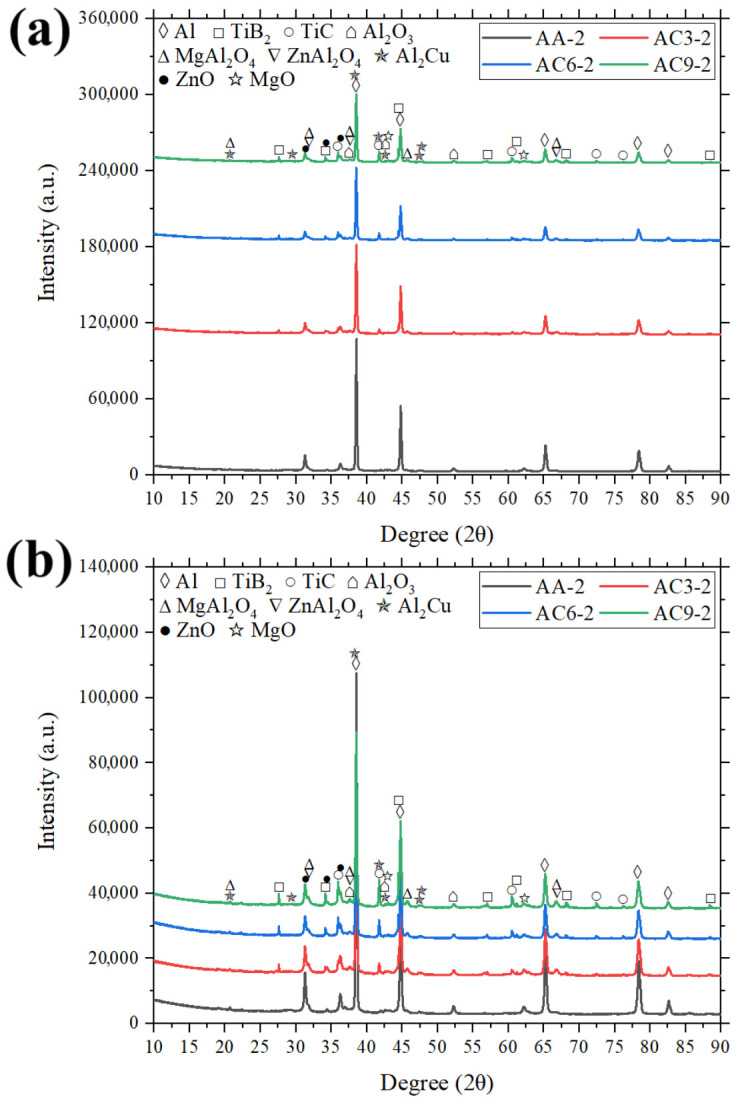
XRD patterns of the samples sintered at 600 °C: (**a**) overall diffraction patterns and (**b**) enlarged low-intensity region for minor peak identification.

Figure 9 and Figure 10 show XRD patterns of sintered samples at 550 and 600 °C, respectively. Phase identification used ICSD 166867 (α-Al), 190893 (TiB_2_), 618944 (TiC), 99783 (α-Al_2_O_3_), 52026 (MgO), 65121 (ZnO), 31373 (MgAl_2_O_4_), and 9559 (ZnAl_2_O_4_). At both temperatures, α-Al remained the dominant matrix phase, while TiB_2_ and TiC reflections in reinforced samples confirmed retention of these ceramic phases after sintering.

The magnified low-intensity regions in Figure 9 and Figure 10 reveal weak reflections from α-Al_2_O_3_, MgO, ZnO, MgAl_2_O_4_, and ZnAl_2_O_4_. These peaks indicate partial retention of oxides present in mechanically alloyed powders and limited spinel formation via interface-controlled reactions, rather than a complete bulk transformation. Possible MgO + Al_2_O_3_ → MgAl_2_O_4_ and ZnO + Al_2_O_3_ → ZnAl_2_O_4_ reactions should therefore be interpreted with caution due to low peak intensities and possible overlap with α-Al, TiB_2_, TiC, oxide, or intermetallic reflections.

The limited formation of low-temperature spinel can be attributed to the combined effects of mechanical alloying and microwave sintering. High-energy milling produces defects, fresh surfaces, close oxide–oxide contact, and short diffusion distances, facilitating solid-state reactions. Mechanical activation has been reported to reduce the formation temperature and activation energy of MgAl_2_O_4_ [73,74], while ZnAl_2_O_4_ can form via mechanochemical or ball-milling-assisted ZnO–Al_2_O_3_/ZnO–Al routes [75,76]. Microwave heating and close-contact alumina-based systems can further promote the formation of Mg/Zn aluminate spinel at relatively low temperatures [77,78]. Thus, the weak spinel peaks observed at 550–600 °C likely reflect mechanochemical activation combined with microwave-assisted thermal effects rather than temperature alone.

At 600 °C, some low-intensity phases became more distinct, likely due to enhanced diffusion and increased interfacial reaction probability. However, the coexistence of MgO, ZnO, and α-Al_2_O_3_, along with spinel-related reflections, indicates incomplete oxide consumption. Therefore, the present XRD data suggest limited MgAl_2_O_4_/ZnAl_2_O_4_ or possible (Zn,Mg)Al_2_O_4_-type mixed-spinel contributions, but not definitive identification of a separate mixed-spinel phase. Overall, α-Al remained the primary matrix phase, TiB_2_/TiC persisted as crystalline reinforcements, and the evolution of oxides/spinels was limited under the applied sintering conditions.

### 3.3. Mechanical Properties

**Figure 11:**
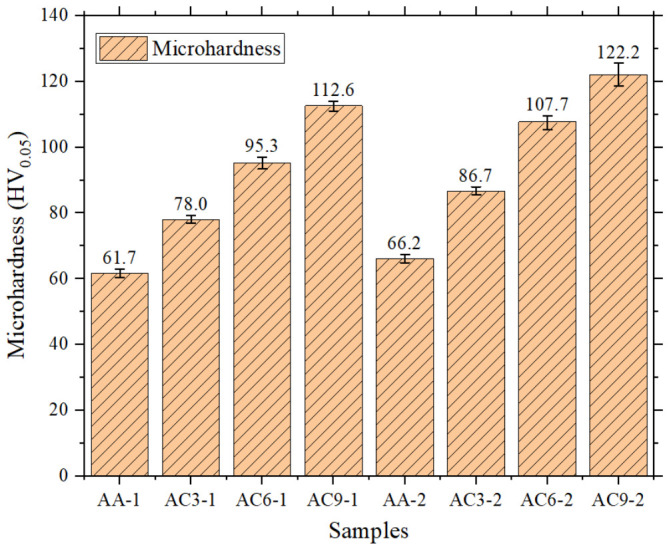
Mean microhardness values of the samples with min–max error bars.

Figure 11 shows average microhardness values, while Table 7 lists the standard deviation and coefficient of variation. Microhardness increased with both TiB_2_/TiC content and sintering temperature. At 550 °C, microhardness rose from 61.7 HV_0.05_ for AA-1 to 78.0, 95.3, and 112.6 HV_0.05_ for AC3-1, AC6-1, and AC9-1. At 600 °C, it increased from 66.2 HV_0.05_ for AA-2 to 86.7, 107.7, and 122.2 HV_0.05_ for AC3-2, AC6-2, and AC9-2. Thus, TiB_2_/TiC hybrid reinforcement markedly improved hardness.

The reinforcement-dependent increase in hardness arises from the combined effects of hard-particle load-bearing, indentation resistance, restricted dislocation motion, crystallite refinement, and lattice microstrain induced by mechanical alloying. The lower crystallite size and higher microstrain in Figure 8 and Table 6 support this interpretation, while previous studies also report hardness improvements associated with TiB_2_ content, milling time, and Al/(TiC+TiB_2_) reinforcement distribution [24,60,63]. Thermal expansion and elastic modulus mismatch between the Al-based matrix and TiB_2_/TiC may further generate residual stresses and geometrically necessary dislocations during cooling, contributing to dislocation hardening under indentation [79,80].

When the effect of sintering temperature is examined, samples sintered at 600 °C exhibit higher microhardness than those sintered at 550 °C for the same composition. For the AA, AC3, AC6, and AC9 samples, the microhardness values increased by approximately 7.3%, 11.2%, 13.0%, and 8.5%, respectively, when the sintering temperature was increased from 550 to 600 °C. This increase is also consistent with the higher $\rho_{rs}$ and φ values reported in Table 5. The higher sintering temperature may have contributed to the formation of a more indentation-resistant microstructure by promoting interparticle bonding, sinter-neck growth, and densification. However, the continued increase in microhardness with increasing reinforcement content, despite the decrease in $\rho_{rs}$ at high TiB_2_/TiC ratios, suggests that the hardness-enhancing effects of the ceramic particles, crystallite refinement, and defect accumulation induced by mechanical alloying became more dominant than the hardness-limiting effect of residual porosity.

The error bars in Figure 11 and coefficient of variation values in Table 7 indicate low microhardness scatter, with coefficients of variation of approximately 1.08–2.06%. The highest scatter in AC9-2 may reflect local effects of reinforcement enrichment, residual porosity, or oxide/secondary phase heterogeneity, consistent with the dark-contrast and Ti-rich regions observed in Figure 4 and Figure 6 [65].

Reported hardness values for AA7068-based composites vary with reinforcement type/content, production route, heat treatment, hardness scale, and indentation load. PM AA7068/MgO, TiO_2_, ZnO, SiC, graphite, and other hybrid composites generally show reinforcement-induced hardening [31,32,33,34,35,37,42,81,82,83,84,85]. Although some reported values are higher, often due to different reinforcements, casting routes, or heat treatment, the maximum value of 122.2 HV_0.05_ obtained here is notable because it was achieved by mechanical alloying–microwave sintering with TiB_2_/TiC reinforcement and without subsequent aging or precipitation hardening. Overall, Figure 11 and Table 7 show that TiB_2_/TiC reinforcement and 600 °C sintering substantially improved microhardness, while possible local heterogeneity at high reinforcement content requires evaluation together with compressive and wear behavior.

**Figure 12:**
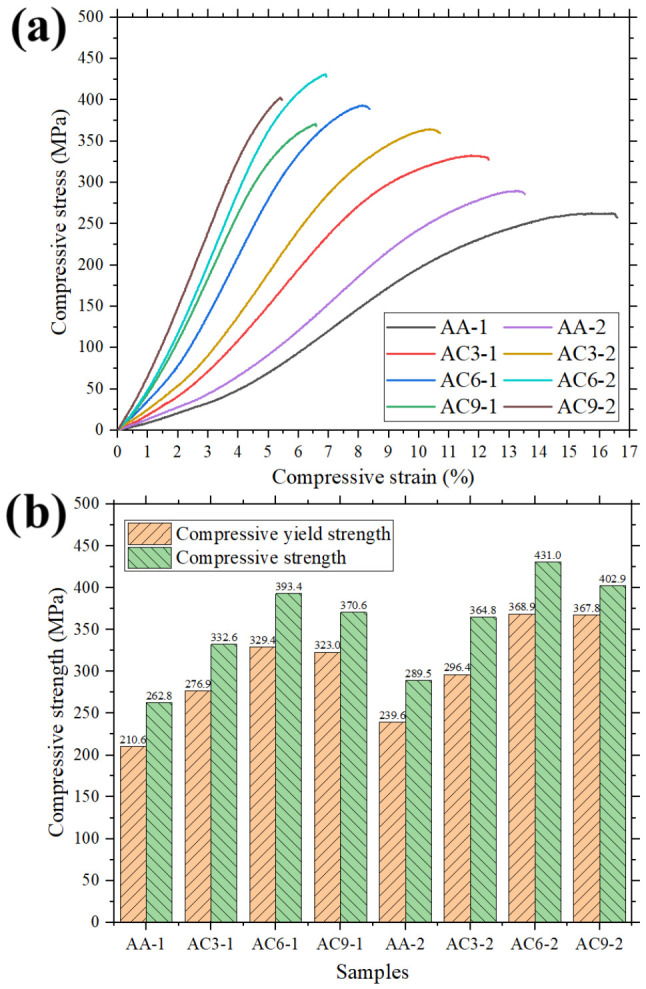
Compression test results of the samples: (**a**) compressive stress–strain curves and (**b**) compressive yield strength and compressive strength values, with the corresponding statistical data provided in Table 8.

Figure 12a shows compressive stress–strain curves; Figure 12b gives compressive yield and maximum compressive strength; and Table 8 lists strength, strain, toughness, and statistical values. Increasing TiB_2_/TiC content markedly increased yield and maximum compressive strength but reduced compressive strain capacity and toughness. This trend reflects load transfer, grain/crystallite refinement, higher dislocation density, hard-particle strengthening, and restricted plastic deformation/dislocation motion in ceramic-reinforced Al composites [24,65].

For the samples sintered at 550 °C, the compressive yield strength increased from 210.55 MPa for AA-1 to a maximum of 329.38 MPa for AC6-1, while the maximum compressive strength increased from 262.75 to 393.38 MPa. At 600 °C, the same trend was observed: the yield strength increased from 239.60 MPa for AA-2 to 368.86 MPa for AC6-2, and the maximum compressive strength increased from 289.48 to 431.05 MPa. These results demonstrate the strong reinforcing effect of TiB_2_/TiC particles on the compressive strength of the 7068-type Al matrix composites.

The strength improvement is also supported by the lower crystallite size and higher lattice microstrain shown in Figure 8 and Table 6. Mechanical alloying-induced defect accumulation, crystallite refinement, and hard-particle dispersion increase load-bearing capacity and deformation resistance [60]. In addition, thermal expansion and elastic modulus mismatch between Al and TiB_2_/TiC during cooling can generate residual stresses and geometrically necessary dislocations at interfaces, increasing dislocation density and strength. However, this strengthening contribution depends on homogeneous particle distribution and sufficient interfacial bonding; agglomeration and residual porosity at high reinforcement contents can limit the net effect [65,79,80].

The highest strength was obtained in AC6 rather than in AC9. AC6-1 and AC6-2 reached maximum compressive strengths of 393.38 and 431.05 MPa, whereas AC9-1 and AC9-2 decreased to 370.63 and 402.89 MPa, respectively. This indicates that load-bearing, dislocation restriction, and matrix deformation limitation dominated up to an optimum reinforcement level, whereas agglomeration, residual porosity, poor local interfacial contact, and stress concentration limited further strengthening at the highest TiB_2_/TiC content. The lower $\rho_{rs}$ and φ of AC9 compared with AC6, together with the local heterogeneity and Ti-rich regions in Figure 4 and Figure 6, support this interpretation. Similar optimum-reinforcement behavior has been reported for ceramic-reinforced Al composites [8,65].

Increasing the sintering temperature from 550 to 600 °C improved both compressive yield strength and maximum compressive strength for all compositions. The maximum compressive strength increased from 262.75 to 289.48 MPa for AA, from 332.64 to 364.76 MPa for AC3, from 393.38 to 431.05 MPa for AC6, and from 370.63 to 402.89 MPa for AC9. This improvement is consistent with enhanced sinter-neck formation, stronger interparticle bonding, and higher $\rho_{rs}$/φ at 600 °C. However, the final mechanical performance remains sensitive to the distribution of reinforcement and residual porosity [66].

Figure 12a and Table 8 also show that deformation capacity decreased with reinforcement content. The compressive strain at maximum stress decreased from 15.73% for AA-1 to 6.56% for AC9-1 and from 13.27% for AA-2 to 5.39% for AC9-2. Similarly, compressive toughness decreased from 24.04 to 14.25 MJ/m^3^ in the 550 °C series and from 19.99 to 12.63 MJ/m^3^ in the 600 °C series. These changes indicate that TiB_2_/TiC reinforcement improves strength but restricts plastic deformation and energy absorption, particularly at high ceramic contents. The coefficients of variation for compressive strength remained within the range of approximately 3.18–4.30%, indicating generally reproducible behavior, with slightly higher scatter at AC9 due to stronger local effects of clustering, porosity, and interfacial heterogeneity.

AA7068-based studies show that ceramic or hybrid reinforcement often increases compressive strength up to an optimum content, beyond which agglomeration, porosity, or weak interfaces may limit strengthening. Kumar et al. [86] reported that the strength of AA7068/Si_3_N_4_ increased from approximately 310 MPa for AA7068 to 395 MPa at 1.5 wt.% Si_3_N_4_, but decreased to 355 MPa at 2 wt.% due to agglomeration. The present AC6-2 value of 431.05 MPa is therefore competitive with, or higher than, those reported for some Si_3_N_4_-reinforced AA7068 nanocomposites. Similarly, Soni and Mandloi [82] reported an increase from 613.52 MPa for AA7068 to 655.54 MPa at 1 wt.% TiB_2_ + 2 wt.% fly ash, followed by a reduction at higher fly ash contents, while Abhilash et al. [29] observed optimum-level strengthening in Al_2_O_3_-reinforced AA7068. These trends support the observed decrease in AC6-to-AC9 strength here and confirm that excessive reinforcement can reduce macro-scale load-bearing capacity through heterogeneity, porosity, weak interfaces, and particle aggregation.

Overall, Figure 12 and Table 8 show that TiB_2_/TiC reinforcement improved compressive yield and maximum strength, with AC6-2 reaching the highest compressive strength of 431.05 MPa. The lower strength of AC9, despite its highest microhardness, confirms that hardness and compressive strength are not linearly coupled; macroscale compression depends strongly on densification, interfacial integrity, and reinforcement distribution homogeneity.

### 3.4. Tribological Behavior

**Figure 13:**
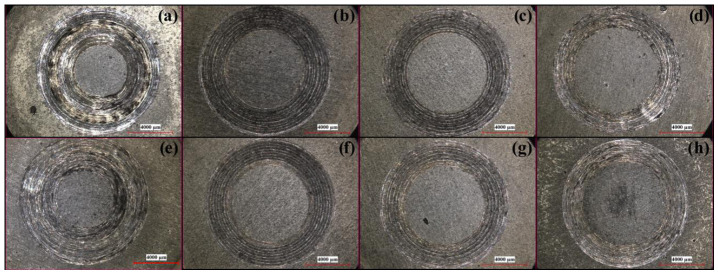
Macroscopic stereomicroscope images of the worn surfaces of the samples after 1000 m of sliding distance: (**a**) AA-1, (**b**) AC3-1, (**c**) AC6-1, (**d**) AC9-1, (**e**) AA-2, (**f**) AC3-2, (**g**) AC6-2, and (**h**) AC9-2.

Figure 13 shows stereomicroscope images of worn surfaces after 1000 m. All samples exhibited circular wear tracks under ball-on-disc contact. AA-1 and AA-2 showed more pronounced, brighter, and locally wider tracks, indicating greater plastic deformation and surface material removal in the unreinforced matrix, especially in AA-1 due to lower hardness and limited load-bearing capacity.

In TiB_2_/TiC-reinforced samples, the wear tracks became more regular and narrower, indicating that ceramic particles restricted matrix plastic deformation, reduced direct Al-based matrix–counterface contact, and improved surface load-bearing capacity. Samples sintered at 600 °C generally showed more integrated and stable wear-track morphologies, consistent with their higher $\rho_{rs}$, φ, and microhardness. However, because macroscopic images alone cannot identify the active wear mechanism, Figure 13 should be interpreted together with wear loss, specific wear rate, friction, and SEM wear-surface analyses.

**Figure 14:**
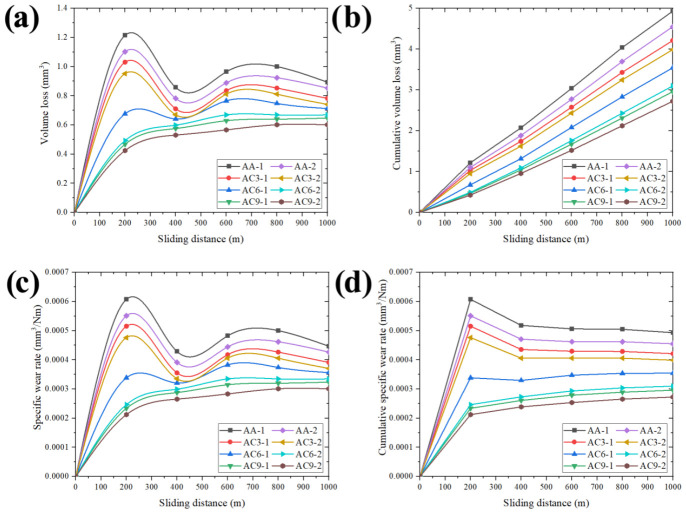
Wear loss behavior of all samples: (**a**) interval volume loss, (**b**) cumulative volume loss, (**c**) interval-specific wear rate, and (**d**) cumulative specific wear rate.

Figure 14 presents interval volume loss, cumulative volume loss, interval-specific wear rate, and cumulative specific wear rate. Interval volume loss is the material loss measured within each sliding-distance interval, whereas cumulative volume loss is the total material loss accumulated up to the corresponding distance. Similarly, the interval-specific wear rate in Figure 14c represents a distance-dependent wear factor calculated for each interval, not the final wear rate of the entire test. In contrast, Figure 14d shows the cumulative specific wear rate, calculated from cumulative volume loss and total sliding distance. Such interval-based wear-factor curves may exhibit higher values and greater fluctuations during running-in due to asperity removal, debris formation, surface adaptation, and transient changes in mechanisms [87]. Cumulative volume loss increased with distance, while interval volume loss and interval-specific wear rate fluctuated but became more stable after running-in.

Cumulative volume loss clearly shows the reinforcement effect. After 1000 m at 550 °C, volume loss decreased from 4.929 mm^3^ for AA-1 to 4.211, 3.543, and 2.959 mm^3^ for AC3-1, AC6-1, and AC9-1, corresponding to reductions of 14.6%, 28.1%, and 40.0%. At 600 °C, it decreased from 4.549 mm^3^ for AA-2 to 3.986, 3.099, and 2.723 mm^3^ for AC3-2, AC6-2, and AC9-2, corresponding to reductions of 12.4%, 31.9%, and 40.1%. Thus, TiB_2_/TiC reinforcement increased load-bearing capacity, reduced direct matrix–counterface contact, and limited material detachment.

Specific wear rate results in Figure 14c,d followed the same trend. During the first 200 m, the interval-specific wear rate decreased progressively with increasing reinforcement content at both sintering temperatures, indicating reduced initial material removal after the addition of TiB_2_/TiC. After 1000 m, the cumulative specific wear rate decreased from 4.929 × 10^−4^ to 2.959 × 10^−4^ mm^3^/Nm at 550 °C and from 4.549 × 10^−4^ to 2.723 × 10^−4^ mm^3^/Nm at 600 °C when AA and AC9 are compared. The lowest value in AC9-2 confirms the highest wear resistance.

Samples sintered at 600 °C generally showed lower cumulative volume loss and specific wear rate than those sintered at 550 °C. After 1000 m, the cumulative volume loss decreased from 4.929 to 4.549 mm^3^ for AA, from 4.211 to 3.986 mm^3^ for AC3, from 3.543 to 3.099 mm^3^ for AC6, and from 2.959 to 2.723 mm^3^ for AC9, corresponding to reductions of approximately 7.7%, 5.3%, 12.5%, and 8.0%, respectively. This improvement is attributed to higher $\rho_{rs}$ and φ, increased microhardness, and stronger interparticle bonding at 600 °C, which reduced particle pull-out, surface material removal, and detachment during sliding wear.

The wear results were also evaluated using the Archard approach, in which wear volume is proportional to normal load and sliding distance and inversely proportional to hardness [88], as given in Equation (7):

(7) $$\begin{array}{c} V=\frac{KFL}{H} \end{array}$$

Here, V is the wear volume, K is the dimensionless Archard wear coefficient, F is the normal load, L is the sliding distance, and H is hardness. The cumulative specific wear rate after 1000 m is given by Equation (8), and combining Equations (7) and (8) yields the Archard coefficient in Equation (9):

(8) $$\begin{array}{c} k_{cum}=\frac{V}{FL} \end{array}$$

(9) $$\begin{array}{c} K=k_{cum}H\times 10^{-3} \end{array}$$

Since hardness was measured as HV_0.05_, it was converted to MPa using Equation (10). Because cumulative specific wear rate is expressed in mm^3^/Nm, the meter-to-millimeter conversion was also included, yielding the final calculation form in Equation (11):

(10) $$\begin{array}{c} H\left(MPa\right)={HV}_{0.05}\times 9.807 \end{array}$$

(11) $$\begin{array}{c} K=k_{cum}\times {HV}_{0.05}\times 9.807\times 10^{-3} \end{array}$$

In Equation (11), 9.807 converts HV to MPa, while 10^−3^ accounts for the meter-to-millimeter conversion. The calculated values are listed in Table 9.

As shown in Table 9, the cumulative specific wear rate decreased markedly with TiB_2_/TiC reinforcement, whereas the dimensionless Archard wear coefficient remained within a narrow range of 2.953 × 10^−4^ to 3.389 × 10^−4^. This indicates that the reduced wear volume was largely associated with increased microhardness, reduced surface penetration, and load-bearing by TiB_2_/TiC. However, the K values did not decrease dramatically, showing that reinforcement distribution, interfacial integrity, residual porosity, and third-body/tribolayer effects also governed the tribological response.

Although consistent with the Archard approach, wear behavior cannot be explained solely by hardness. TiB_2_/TiC particles reduce matrix–counterface contact, while oxidized and compacted wear debris can form transient tribolayers or mechanically mixed layers. Stable layers limit material loss, whereas local fracture, detachment, or renewal causes fluctuations in volume loss and specific wear rate. Therefore, the initially high wear loss and subsequent stabilization observed in Figure 14a,c should be interpreted in terms of running-in, tribolayer formation, and layer stability [2,37,65]. Figure 14 and Table 9 confirm that TiB_2_/TiC reinforcement and 600 °C sintering reduced wear loss, with AC9-2 showing the best resistance.

**Figure 15:**
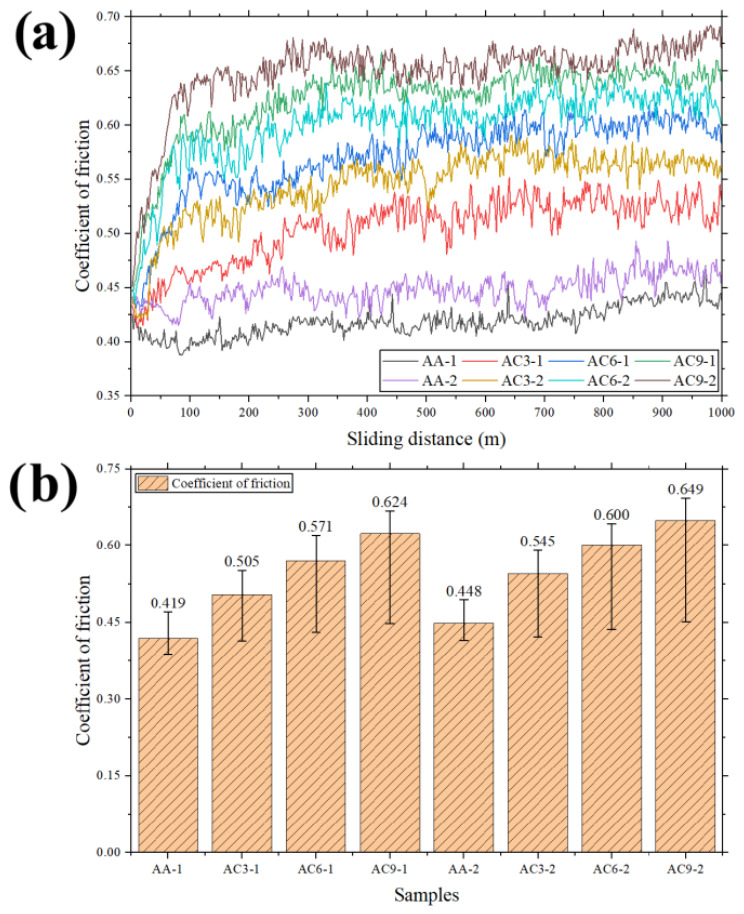
Friction behavior of all samples: (**a**) continuous COF curves recorded from 0 to 1000 m, and (**b**) average COF values with min–max error bars.

Figure 15a shows friction coefficient curves over 0–1000 m, while Figure 15b presents average values with min–max error bars. The coefficient of friction (COF) increased rapidly at the onset of sliding and then entered a fluctuating yet relatively stable regime, reflecting asperity removal, local plastic deformation, debris formation, compaction, debris removal, and third-body action during dry sliding.

The average COF increased with TiB_2_/TiC content. At 550 °C, it increased from 0.419 for AA-1 to 0.624 for AC9-1, while at 600 °C it increased from 0.448 for AA-2 to 0.649 for AC9-2. Thus, TiB_2_/TiC reinforcement reduced wear loss but increased friction, indicating that wear resistance and friction are governed by related yet distinct surface processes. Hard ceramic particles restrict matrix deformation and material removal, but when exposed or detached, they can increase shear resistance, micro-ploughing, roughness interaction, and third-body abrasion. Similar Al composite studies report that hard particles improve load-bearing and wear resistance while friction remains sensitive to particle protrusion, debris, roughness, and third-body interactions [2,37,65].

The higher COF at 600 °C for each composition may be related to higher density, stronger interparticle bonding, and higher microhardness. A denser and harder surface reduces material removal but can increase shear resistance in hard-contact regions. Therefore, the best wear resistance in AC9-2 should be assessed by wear loss, surface damage, third-body effects, and wear mechanisms rather than by a low friction coefficient.

Continuous friction curves show that AA samples had lower average friction but higher wear loss, whereas AC9-1 and AC9-2 had the highest friction yet the lowest wear loss. This indicates that high TiB_2_/TiC content increased surface load-bearing capacity and reduced material removal, while increasing shear resistance. The highest friction in AC9-2 is attributed to the combined effects of high ceramic content, higher microhardness, and stronger interparticle bonding at 600 °C. Thus, wear resistance in this system is mainly linked to hardness, hard-phase load-bearing capacity, densification, and limited material detachment rather than to low friction.

**Figure 16:**
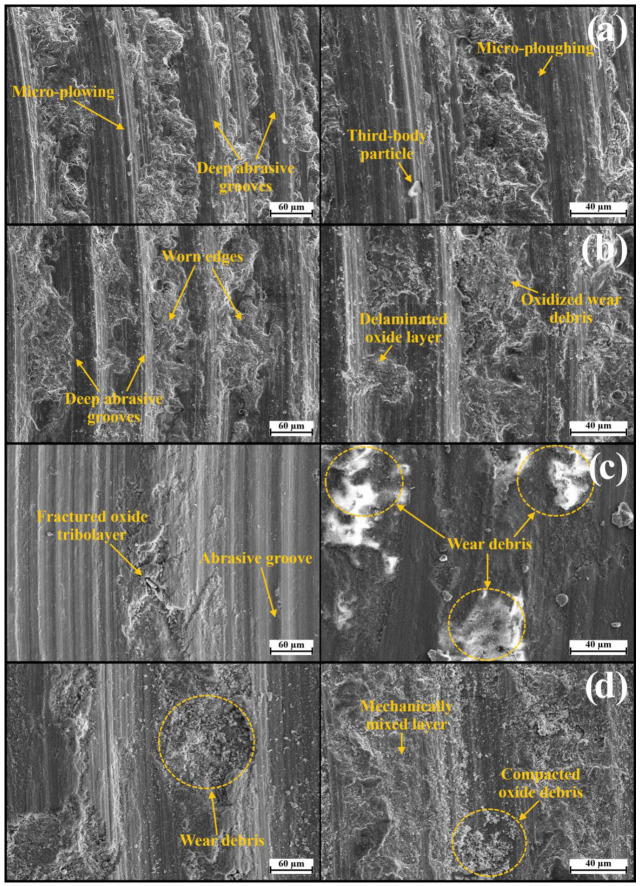
SEM micrographs of the worn surfaces of the samples sintered at 550 °C at low and high magnifications: (**a**) AA-1, (**b**) AC3-1, (**c**) AC6-1, and (**d**) AC9-1.

**Figure 17:**
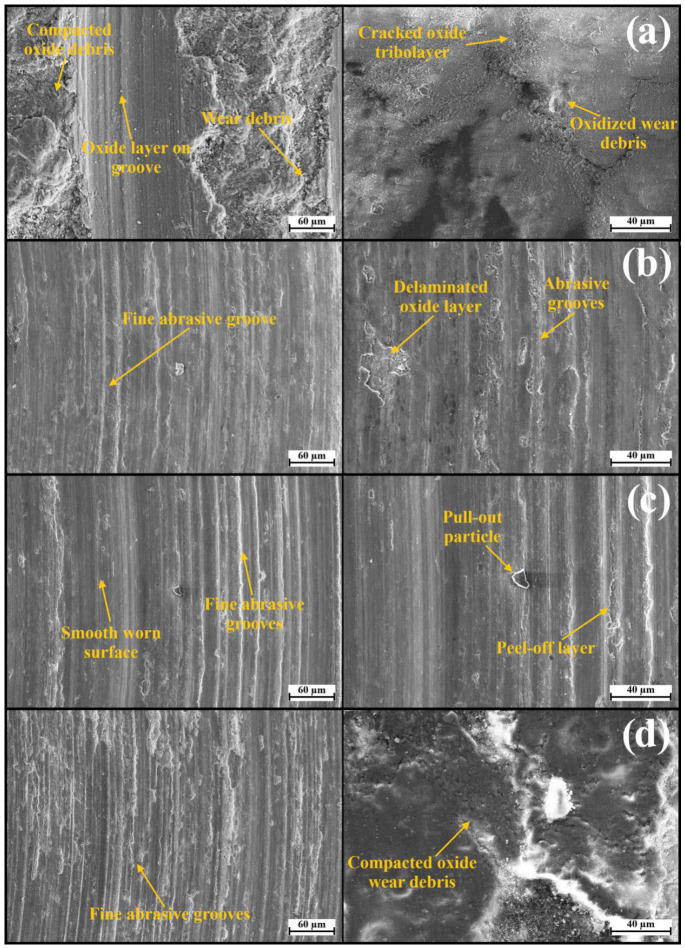
SEM micrographs of the worn surfaces of the samples sintered at 600 °C at low and high magnifications: (**a**) AA-2, (**b**) AC3-2, (**c**) AC6-2, and (**d**) AC9-2.

SEM wear-surface observations in Figure 16 and Figure 17 further clarify the mechanisms suggested by Figure 13, Figure 14 and Figure 15. AA-1 exhibited deep grooves, micro-ploughing, localized third-body particles, and severe plastic flow, consistent with its highest wear loss and low hardness. In AC3-1, deep grooves and locally delaminated oxide layers persisted, indicating partial improvement in wear resistance but unstable tribological layers at low reinforcement content. AC6-1 showed reduced groove depth and more regular tracks. At the same time, AC9-1 exhibited more pronounced mechanically mixed layers and compacted oxide-rich debris, indicating a more complex third-body/tribolayer structure containing ceramic fragments, oxidized debris, and Al-based matrix components.

At 600 °C, the worn surfaces generally became more integrated. AA-2 showed compacted oxide-rich debris and cracked tribolayer regions but remained unreinforced and therefore susceptible to deformation. AC3-2 displayed fine grooves and locally delaminated oxide layers, whereas AC6-2 exhibited a smoother surface, fine grooves, limited particle detachment, and local peel-off layers, consistent with its balanced density, hardness, and compressive strength. AC9-2 showed fine grooves and compacted oxide-rich/dense tribolayers, consistent with its lowest wear loss.

Together, Figure 16 and Figure 17 show that increasing reinforcement content and sintering temperature shifted wear from severe micro-ploughing and deep grooves toward finer grooves, compacted tribolayers, oxidized debris, and mechanically mixed layers. TiB_2_/TiC particles restricted matrix deformation and increased surface load-bearing capacity, while particle detachment, debris accumulation, and third-body effects persisted at high reinforcement contents, thereby contributing to higher friction. This also explains why AC9-2 exhibited the best wear resistance, whereas AC6-2 showed the highest compressive strength: surface load-bearing and tribolayer stability dominate wear resistance, whereas porosity, interfacial integrity, and reinforcement heterogeneity govern compressive strength.

**Figure 18:**
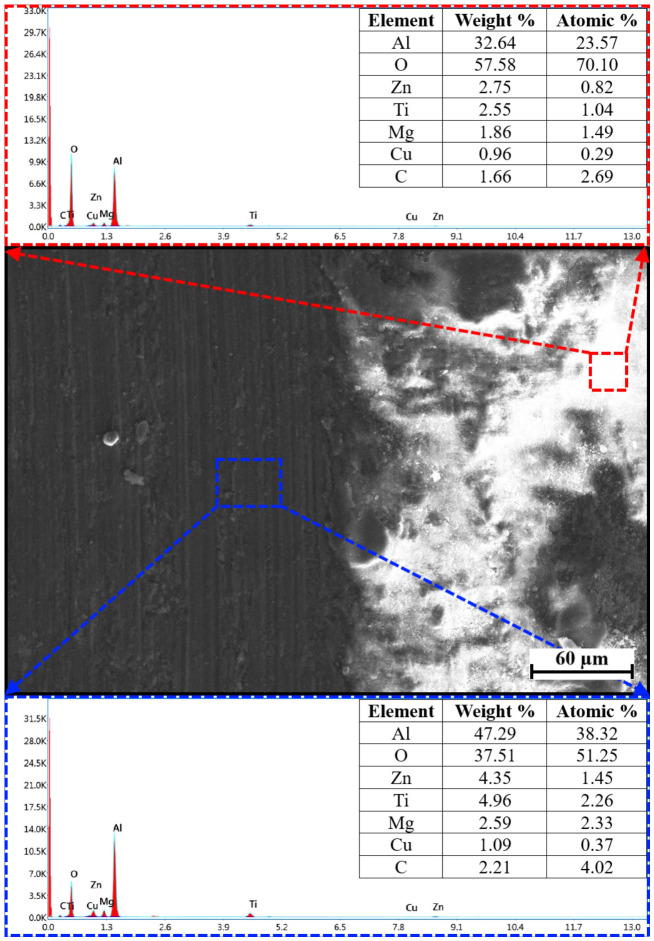
SEM image and corresponding EDS analyses of worn regions with relatively low and high oxygen contents on AC3-1.

**Figure 19:**
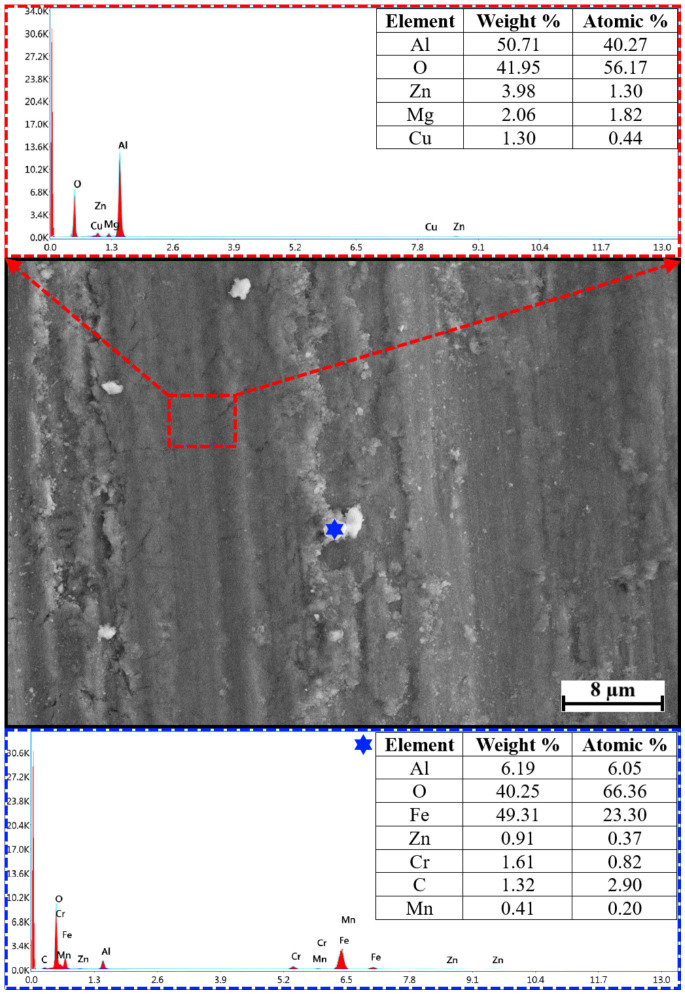
SEM image and corresponding EDS analyses of a worn-surface region and an Fe-rich particle on AC6-2.

**Figure 20:**
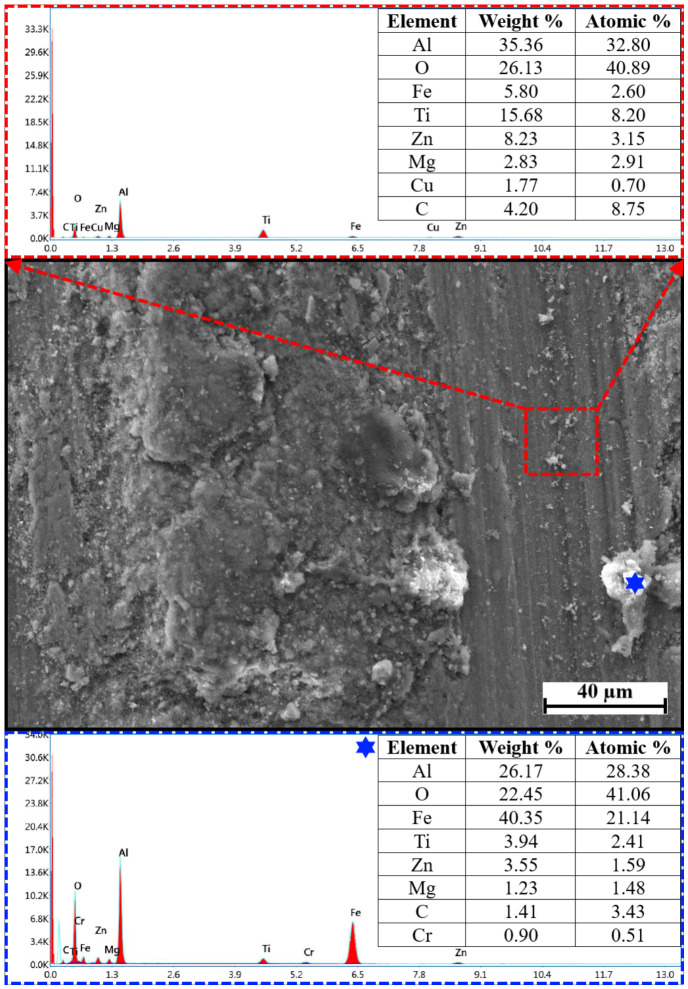
SEM image and corresponding EDS analyses of a worn-surface region and an Fe-rich particle on AC9-1.

SEM–EDS analyses in Figure 18, Figure 19 and Figure 20 provide chemical evidence for the presence of oxide-rich tribolayers, TiB_2_/TiC-derived fragments, and third-body debris. In Figure 18, AC3-1 showed oxygen-rich and lower-oxygen regions containing 57.58 wt.% O/32.64 wt.% Al and 37.51 wt.% O/47.29 wt.% Al, respectively. These results indicate heterogeneous Al-based oxide layers and/or trapped oxide-rich debris affected by local fracture, detachment, and recompression. Ti signals in both regions, including 4.96 wt.% Ti in the lower-oxygen region, suggest exposed or mechanically incorporated TiB_2_/TiC-derived particles, although B and C quantification remains only supportive due to their low atomic numbers [67].

For AC6-2 (Figure 19), the worn surface contained 50.71 wt.% Al and 41.95 wt.% O, indicating an Al-based oxidized tribolayer that may have reduced direct metal-to-metal contact and limited wear loss. The star-marked particle contained 49.31 wt.% Fe and 40.25 wt.% O with minor Cr and Mn, suggesting oxidized steel-derived transfer or an Fe-rich third-body particle. Such particles can contribute to the formation of an oxide tribolayer and reduce material loss, while also increasing local micro-abrasive interactions and friction.

For AC9-1 (Figure 20), the high Ti content of 15.68 wt.% and C content of 4.20 wt.% indicate a greater contribution from ceramic particles/fragments to the mechanically mixed tribological layer, while O at 26.13 wt.% reflects oxidized Al–Zn–Mg-based debris. The Fe-rich particle containing 40.35 wt.% Fe with Al, O, Ti, Zn, and Cr suggests a mechanically mixed third-body product with steel-derived transfer. Together, Figure 18, Figure 19 and Figure 20 confirm that the improved wear resistance of TiB_2_/TiC composites resulted from hard-particle load-bearing, oxide/tribolayer formation, and third-body effects rather than hardness alone.

**Figure 21:**
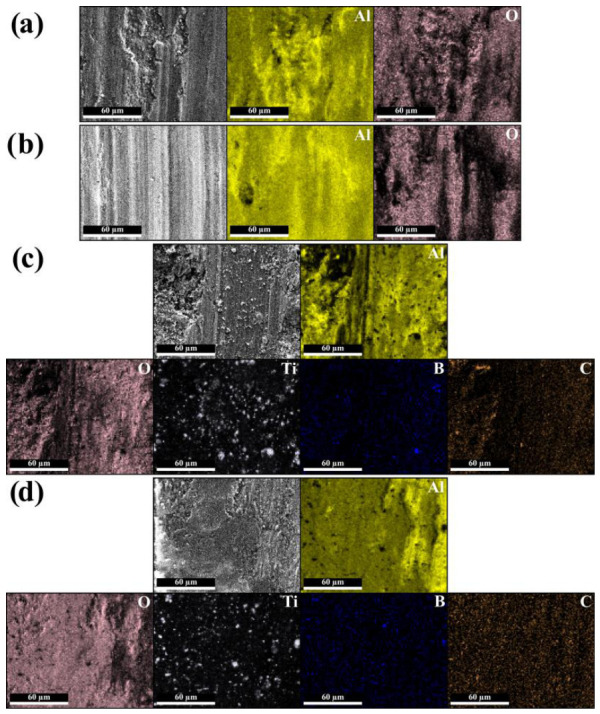
SEM image and corresponding elemental mapping results of the worn samples: (**a**) AA-1, (**b**) AA-2, (**c**) AC9-1, and (**d**) AC9-2.

Figure 21 compares elemental maps of AA-1, AA-2, AC9-1, and AC9-2 after wear. In AA-1 and AA-2, Al dominated the tracks, while O-rich regions appeared along the grooves and in damaged areas, indicating the formation of Al-based oxide/debris. AA-2 showed more regular grooves and more continuous oxygen-containing regions, likely due to improved densification and bonding at 600 °C. However, since AA samples contain no TiB_2_/TiC, their wear resistance was governed mainly by matrix hardness, oxide-layer stability, and debris behavior; their high volume loss despite low friction reflects easy plastic deformation of the soft Al-based matrix.

In AC9-1 and AC9-2, localized Ti-rich particles with weaker B and C signals indicate the retention or exposure of TiB_2_/TiC particles on the worn surface, although quantitative B/C assessment remains uncertain [67]. The O signal along the tracks indicates a mechanically mixed tribological layer containing oxidized debris and ceramic particles, consistent with the oxygen-rich, Ti-containing, and Fe-rich third-body features in Figure 18, Figure 19 and Figure 20. Compared with AC9-1, AC9-2 exhibited more continuous Ti-rich particles and Al–O-containing wear layers, suggesting that 600 °C sintering improved densification and matrix–reinforcement contact. This helped ceramic particles remain stable as load-bearing phases during wear and is consistent with the lowest volume loss and cumulative specific wear rate of AC9-2.

AA7068 wear studies show that reinforcement type, production route, heat treatment, load, and sliding distance strongly control wear response. Literature values generally support reinforcement-induced wear reduction: in situ AA7068–ZrB_2_ composites decreased from about 5.3 × 10^−4^ mm^3^/Nm for unreinforced AA7068 to 2.1 × 10^−4^ mm^3^/Nm at 9 vol.% ZrB_2_ [36], while HEA/MEA- and hybrid-reinforced AA7068 systems also showed reduced specific wear rates depending on reinforcement chemistry and content [41,42,43,44,45]. PM AA7068 composites reinforced with ZnO, SiC, graphite, TiC, Si_3_N_4_, and GNPs similarly exhibit a decreasing wear rate with the addition of reinforcements, mainly due to increased hardness, load-bearing capacity, and improved surface stability [2,30,33,34,35,37].

Using mass-loss values extracted from Naganambi and Selvakumar [42], AA7068/graphene–TiC composites decreased from about 5.61 × 10^−4^ mm^3^/Nm for cast AA7068 to 4.85 × 10^−4^ mm^3^/Nm with 1.0 wt.% graphene, and further to 2.38 × 10^−4^ mm^3^/Nm for the highest graphene–TiC condition. The present AC9-2 and AC9-1 values of 2.723 × 10^−4^ and 2.959 × 10^−4^ mm^3^/Nm are therefore within the same order of magnitude as the lower wear rates reported for AA7068 composites, demonstrating competitive wear resistance of TiB_2_/TiC reinforcement produced by mechanical alloying and microwave sintering.

Some AA7068 studies report wear loss, depth, or rate without sufficiently defined volumetric units and/or normal load; therefore, they are compared only on a trend basis. These studies show that artificial aging, ZrO_2_ reinforcement, Al_2_O_3_ reinforcement with aging/cryogenic treatment, and load/speed changes can significantly alter the wear regimes of AA7068 [29,38,39,40]. Overall, the low cumulative specific wear rates obtained here are linked to higher microhardness, ceramic load-bearing capacity, reduced surface penetration, and oxide/tribolayer-supported third-body contact, highlighting the distinctive contribution of the TiB_2_/TiC hybrid reinforcement under the present processing route.

## 4. Conclusions

This study produced 7068-type Al–Zn–Mg–Cu-based matrix alloy and TiB_2_/TiC hybrid-reinforced composites via mechanical alloying and microwave sintering. Reinforcement content and sintering temperature were evaluated with respect to powder morphology, densification, phase formation, microstructure, microhardness, compression, and dry sliding wear. The main findings are summarized as follows:

➢ Mechanical alloying refined the powders: D_50_ decreased from 51.5 µm for AA to 22.5 µm for AC9, and D[4,3] decreased from 125 to 32.1 µm, confirming the fracture-inducing role of TiB_2_/TiC particles.
➢ Increasing the sintering temperature from 550 to 600 °C improved interparticle bonding, microstructural integrity, and densification, although local reinforcement enrichment and residual porosity remained in high-TiB_2_/TiC AC9 samples.
➢ XRD confirmed α-Al as the primary matrix phase, retained TiB_2_/TiC phases, and low-intensity MgAl_2_O_4_/ZnAl_2_O_4_ spinel-type phases, indicating limited interfacial reactions associated with milling-induced contact, processing oxides, and microwave sintering.
➢ W–H and SSP analyses showed crystallite refinement and increased lattice microstrain with TiB_2_/TiC addition. W–H values changed from 24.03 nm and 0.1130% for AA to 20.07 nm and 0.1380% for AC9, while SSP values changed from 29.51 nm and 0.2221% to 23.94 nm and 0.2945%.
➢ Microhardness increased from 61.7 HV_0.05_ for AA-1 to 122.2 HV_0.05_ for AC9-2 due to hard-particle load-bearing, milling-induced defect/crystallite structure, possible thermal-mismatch dislocations, and improved bonding at 600 °C.
➢ TiB_2_/TiC reinforcement increased compressive strength, with the highest value of 431.05 MPa in AC6-2. The lower strength of AC9-2, despite its higher hardness, shows that compression depends on densification, porosity, interfacial bonding, and heterogeneity in reinforcement distribution, not hardness alone.
➢ Wear resistance improved with reinforcement. After 1000 m, cumulative volume loss decreased from 4.929 mm^3^ for AA-1 to 2.723 mm^3^ for AC9-2, which also exhibited the lowest cumulative specific wear rate of 2.723 × 10^−4^ mm^3^/Nm. This improvement resulted from reduced penetration, increased load-bearing, and limited Al matrix–counterface contact.
➢ Archard analysis showed that the wear coefficient remained within the same order of magnitude, ranging from 2.953 × 10^−4^ to 3.389 × 10^−4^. Thus, wear reduction was mainly hardness-controlled but also affected by reinforcement distribution, interfacial integrity, porosity, and tribolayer/third-body effects.
➢ The COF increased with TiB_2_/TiC content, reaching 0.649 for AC9-2 compared with 0.419 for AA-1. This is consistent with lower wear loss, as hard particles and trapped debris reduce material removal while increasing shear resistance and micro-abrasive/third-body interactions.
➢ SEM–EDS observations showed severe plastic deformation, deep grooves, and discontinuous oxide layers in unreinforced samples, whereas TiB_2_/TiC composites exhibited finer grooves, oxidized debris, mechanically mixed layers, and Fe-rich third-body particles, explaining the simultaneous reduction in wear loss and increase in friction.

Overall, mechanical alloying combined with microwave sintering is effective for producing TiB_2_/TiC hybrid-reinforced 7068-type Al matrix composites. AC6-2 provided the most balanced compressive-strength response, whereas AC9-2 delivered the highest hardness and wear resistance. Future studies should focus on artificial aging/T6-T7 treatments, retrogression and re-aging, deep cryogenic treatment [29,40], corrosion/tribocorrosion behavior [89], wear mechanism maps [38], and advanced interface characterization to clarify engineering applicability.

## Figures and Tables

**Table 1:** Target chemical composition of the 7068-type Al–Zn–Mg–Cu-based matrix alloy (wt.%).

Al	Zn	Mg	Cu	Fe	Zr	Si	Mn	Ti	Cr
Balance	8.3	3	2.4	0.15	0.15	0.12	0.1	0.1	0.05

**Table 2:** Nomenclature, chemical compositions (vol.%), and sintering temperatures of the investigated material groups.

Samples	Sintering Temperature (°C)	AA7068	TiB_2_	TiC
AA-1	550	100	–	–
AC3-1		97	1.5	1.5
AC6-1		94	3	3
AC9-1		91	4.5	4.5
AA-2	600	100	–	–
AC3-2		97	1.5	1.5
AC6-2		94	3	3
AC9-2		91	4.5	4.5

**Table 3:** Particle size distribution values (D_10_–D_90_) and true/solid densities of the as-received alloy and reinforcement powders.

Powders	D_10_	D_20_	D_30_	D_40_	D_50_	D_60_	D_70_	D_80_	D_90_	True/Solid Density (g/cm^3^)
**Al**	22.8	30.3	37.1	44.3	52.7	63.1	76.7	96.3	128	2.70
**TiB_2_**	4.10	5.99	7.76	9.65	11.8	14.5	17.9	23.0	31.7	4.52
**TiC**	1.21	2.31	3.91	5.80	7.74	9.85	12.4	16.0	23.2	4.93
**Zn**	3.95	5.23	6.35	7.49	8.73	10.2	12.0	14.4	18.5	7.14
**Mg**	24.8	30.5	35.4	40.1	44.9	50.2	56.3	63.9	74.9	1.74
**Cu**	8.93	11.5	13.8	16.1	18.6	21.4	24.8	29.3	36.5	8.96
**Fe**	15.1	21.0	26.6	33.7	43.3	55.0	67.7	82.1	102	7.87
**Zr**	1.20	2.85	5.54	8.54	11.5	14.6	18.3	23.0	30.7	6.52
**Si**	2.71	4.09	5.46	6.93	8.61	10.6	13.2	16.9	23.5	2.33
**Mn**	5.75	8.55	11.0	13.3	15.9	18.7	22.1	26.6	33.8	7.21
**Ti**	15.3	20.9	25.5	29.9	34.4	39.3	44.9	52.0	62.7	4.51
**Cr**	4.12	6.97	9.43	11.7	13.9	16.2	18.9	22.4	27.4	7.19

**Table 4:** Particle size distribution values of the mechanically alloyed powders, including D[3,2] (Sauter mean diameter) and D[4,3] (De Brouckere mean diameter).

Samples	D_10_	D_50_	D_90_	D[3,2]	D[4,3]
AA	18.6	51.5	130	35.7	125
AC3	9.35	33.9	111	19.2	71
AC6	7.96	27.8	79.1	16.5	36.6
AC9	5.88	22.5	58.8	14.7	32.1

**Table 5:** Density and sinterability values of samples fabricated by PM method.

Samples	$\rho_{a}$ (g/cm^3^)	$\rho_{ra}$ (%)	$\rho_{g}$ (g/cm^3^)	$\rho_{rg}$ (%)	As Sintered at 550 °C		As Sintered at 600 °C		$\rho_{t}$ (g/cm^3^)	φ	
					$\rho_{s}$ (g/cm^3^)	$\rho_{rs}$ (%)	$\rho_{s}$ (g/cm^3^)	$\rho_{rs}$ (%)		550 °C	600 °C
AA	0.5950	20.83	2.6647	93.29	2.7997	98.01	2.8138	98.51	2.8564	0.7042	0.7780
AC3	0.6297	21.62	2.6587	91.29	2.8139	96.61	2.8346	97.33	2.9125	0.6118	0.6934
AC6	0.7676	25.86	2.6867	90.51	2.8086	94.61	2.8399	95.67	2.9685	0.4327	0.5438
AC9	0.8498	28.10	2.7061	89.47	2.7814	91.96	2.8273	93.48	3.0246	0.2364	0.3806

**Table 6:** Comparison of crystallite size and lattice microstrain values obtained from Williamson–Hall and Size–Strain Plot analyses.

Samples	Williamson-Hall		Size–Strain Plot	
	Crystallite Size (nm)	Lattice Microstrain (%)	Crystallite Size (nm)	Lattice Microstrain (%)
AA	24.03	0.1130	29.51	0.2221
AC3	20.66	0.1330	25.50	0.2578
AC6	20.21	0.1370	25.21	0.2637
AC9	20.07	0.1380	23.94	0.2945

**Table 7:** Statistical summary of microhardness measurements of the produced samples.

Sample	Microhardness (HV_0.05_)	Standard Deviation (HV_0.05_)	Coefficient of Variation (%)
AA-1	61.7	1.15	1.86
AC3-1	78.0	0.94	1.20
AC6-1	95.3	1.57	1.65
AC9-1	112.6	1.22	1.08
AA-2	66.2	1.00	1.51
AC3-2	86.7	1.04	1.20
AC6-2	107.7	1.78	1.65
AC9-2	122.2	2.52	2.06

**Table 8:** Compressive properties, strain parameters, toughness, and statistical summary of the produced samples.

Sample	Compressive Yield Strength (MPa)	Compressive Strength (MPa)	Statistical Data of Compressive Strength		Compressive Fracture Strength (MPa)	Compressive Yield Point Strain (%)	Compressive Strength Point Strain (%)	Compressive Fracture Point Strain (%)	Compressive Toughness (MJ/m^3^)
			Standard Deviation (MPa)	Coefficient of Variation (%)					
AA-1	210.55	262.75	8.9	3.38	257.52	10.72	15.73	16.59	24.04
AC3-1	276.88	332.64	12.2	3.70	327.35	8.16	11.73	12.32	23.09
AC6-1	329.38	393.38	13.6	3.42	388.95	5.90	8.12	8.36	20.29
AC9-1	323.05	370.63	15.9	4.30	368.13	5.00	6.56	6.59	14.25
AA-2	239.60	289.48	11.2	3.84	285.82	9.85	13.27	13.52	19.99
AC3-2	296.36	364.76	11.7	3.18	359.63	7.27	10.35	10.71	21.32
AC6-2	368.86	431.05	17.2	3.96	428.14	5.12	6.89	6.92	16.12
AC9-2	367.79	402.89	14.9	3.70	399.94	4.61	5.39	5.44	12.63

**Table 9:** Archard wear coefficient calculated from cumulative specific wear loss and microhardness values at 1000 m sliding distance.

Samples	$k_{cum}$ at 1000 m (×10^−4^ mm^3^/Nm)	HV_0.05_	H (MPa)	K (×10^−4^)
AA-1	4.929	61.7	605.09	2.983
AC3-1	4.211	78.0	764.95	3.221
AC6-1	3.543	95.3	934.61	3.311
AC9-1	2.959	112.6	1104.27	3.268
AA-2	4.549	66.2	649.22	2.953
AC3-2	3.986	86.7	850.27	3.389
AC6-2	3.099	107.7	1056.21	3.273
AC9-2	2.723	122.2	1198.42	3.264

## Data Availability

The original contributions presented in this study are included in the article. Further inquiries can be directed to the corresponding author.

## References

[1] Kim M.-S., Kim J. (2024). Development of Low-Pressure Die-Cast Al−Zn−Mg−Cu Alloy Propellers—Part I: Hot Tearing Simulations for Alloy Optimization. Materials.

[2] Kumar A., Rana R.S., Purohit R., Saxena K.K., Xu J., Malik V. (2022). Metallographic Study and Sliding Wear Optimization of Nano Si_3_N_4_ Reinforced High-Strength Al Metal Matrix Composites. Lubricants.

[3] Bindu M.D., Tide P.S., Bhasi A.B., Ramachandran K.K. (2021). Modeling and parametric optimization of friction stir welding of aluminium alloy AA7068-T6 using response surface methodology and desirability function analysis. Bull. Pol. Acad. Sci. Tech. Sci..

[4] Lakshmipathy J., Rajesh Kannan S., Manisekar K., Vinoth Kumar S. (2017). Effect of Reinforcement and Tribological Behaviour of AA 7068 Hybrid Composites Manufactured through Powder Metallurgy Techniques. Appl. Mech. Mater..

[5] Kumar P., Srivastava V.K., Sharma A. (2025). Influence of Cooling Mediums on Mechanical and Tribological Characteristics of Al/Cu-Based Composites Reinforced with Chromium Particles. Tribol. Mater..

[6] Vencl A., Rac A., Bobić I., Mišković Z. (2006). Tribological Properties of Al-Si Alloy A356 Reinforced with Al_2_O_3_ Particles. Tribol. Ind..

[7] Vencl A., Bobić I., Jovanović M.T., Babić M., Mitrović S. (2008). Microstructural and Tribological Properties of A356 Al–Si Alloy Reinforced with Al_2_O_3_ Particles. Tribol. Lett..

[8] Fanani E.W.A., Surojo E., Prabowo A.R., Akbar H.I. (2021). Recent Progress in Hybrid Aluminum Composite: Manufacturing and Application. Metals.

[9] Ervina Efzan M.N., Siti Syazwani N., Mustafa Al Bakri A.M. (2016). Fabrication Method of Aluminum Matrix Composite (AMCs): A Review. Key Eng. Mater..

[10] Vani V.V., Chak S.K. (2018). The Effect of Process Parameters in Aluminum Metal Matrix Composites with Powder Metallurgy. Manuf. Rev..

[11] Sekar B.K., Pradeep G.V.K., Silambarasan R., Dhairiyasamy R. (2024). Microstructural and Mechanical Characterization of AA2124 Aluminum Alloy Matrix Composites Reinforced with Si_3_N_4_ Particulates Fabricated by Powder Metallurgy and High-Energy Ball Milling. Matéria (Rio J.).

[12] Toozandehjani M., Matori K.A., Ostovan F., Abdul Aziz S., Mamat M.S. (2017). Effect of Milling Time on the Microstructure, Physical and Mechanical Properties of Al-Al_2_O_3_ Nanocomposite Synthesized by Ball Milling and Powder Metallurgy. Materials.

[13] Mondal S., Mondal P., Mishra D.P. (2023). Research Progress on Ceramic Nanomaterials Reinforced Aluminum Matrix Nanocomposites. Mater. Sci. Technol..

[14] Nayak K.C., Rane K.K., Date P.P., Srivatsan T.S. (2022). Synthesis of an Aluminum Alloy Metal Matrix Composite Using Powder Metallurgy: Role of Sintering Parameters. Appl. Sci..

[15] Parveez B., Maleque M.A., Jamal N.A. (2021). Influence of Agro-Based Reinforcements on the Properties of Aluminum Matrix Composites: A Systematic Review. J. Mater. Sci..

[16] Pul M., Dağ İ.E., Şimşek T., Avar B., Chattopadhyay A.K. (2025). Nanocrystalline NiTiB Reinforced Aluminum Matrix Composites: Synthesis, Structural, Mechanical and Corrosion Properties. Proc. Inst. Mech. Eng. Part L J. Mater. Des. Appl..

[17] Navaneethakrishnan K., Veeramani A., Chigilipalli B.K., Cheepu M. (2024). Synthesis and Characterization of Recycled-TiC Reinforced AlZnMgCu Powder Metallurgy Composites. Materials.

[18] Samal P., Vundavilli P.R., Meher A., Mahapatra M.M. (2019). Influence of TiC on Dry Sliding Wear and Mechanical Properties of in Situ Synthesized AA5052 Metal Matrix Composites. J. Compos. Mater..

[19] Velishala M., Pandiripalli M., Chilamban V. (2022). Investigation on Corrosion and Wear Properties of Al-7075/TiC Composites Fabricated by Stir Casting Route. Metall. Mater. Eng..

[20] Sayuti M., Sulaiman S., Baharudin B.T.H.T., Arifin M.K.A. (2016). Metal Matrix Composite Products by Vibration Casting Method. Reference Module in Materials Science and Materials Engineering.

[21] Uday K.N., Rajamurugan G. (2022). Effect of Stir Casting Parameters and Mono/Hybrid Reinforcements on Aluminium Metal Matrix Composite—A Review. Proc. Inst. Mech. Eng. Part C J. Mech. Eng. Sci..

[22] Nie J., Wang F., Li Y., Cao Y., Liu X., Zhao Y., Zhu Y. (2017). Microstructure Evolution and Mechanical Properties of Al-TiB_2_/TiC in Situ Aluminum-Based Composites During Accumulative Roll Bonding (ARB) Process. Materials.

[23] Nie J., Wang F., Chen Y., Mao Q., Yang H., Song Z., Liu X., Zhao Y. (2019). Microstructure and Corrosion Behavior of Al-TiB_2_/TiC Composites Processed by Hot Rolling. Results Phys..

[24] Hadian M., Shahrajabian H., Rafiei M. (2019). Mechanical Properties and Microstructure of Al/(TiC+TiB_2_) Composite Fabricated by Spark Plasma Sintering. Ceram. Int..

[25] Mirbagheri S.M., Shahrajabian H., Rafiei M. (2022). Effects of Graphene Nanoplatelets on the Microstructure, Mechanical Properties, and Corrosion Behavior of Spark Plasma Sintered Al + 20 Vol.% (TiC + TiB_2_) Hybrid Composites. J. Mater. Eng. Perform..

[26] Ravnikar D., Trdan U., Nagode A., Šturm R. (2020). Energy Density Effect of Laser Alloyed TiB_2_/TiC/Al Composite Coatings on LMZ/HAZ, Mechanical and Corrosion Properties. Metals.

[27] Bai Y., Wei J., Lei N., Li J., Guo Y., Liu M. (2021). Effect of VN and TiB_2_-TiC_x_ Reinforcement on Wear Behavior of Al 7075-Based Composites. Materials.

[28] Cheng Y., Xu J., Yu L., Hu Y., Huang T., Zhang H. (2021). Effect of TiC/TiC–TiB_2_ on Microstructure and Mechanical Properties of Spray Formed 7055 Aluminum Alloy TIG Welded Joints. J. Mater. Res. Technol..

[29] Abhilash S.G., Arpitha G.R., Raghu M.J., Manikanda Prabu N., Hemanth Kumar M. (2025). Influence of Deep Cryogenic and Natural Ageing Treatment on Al 7068- Al_2_O_3_ Metal Matrix Composites. Adv. Mater. Process. Technol..

[30] Alipour M., Eslami-Farsani R. (2017). Synthesis and Characterization of Graphene Nanoplatelets Reinforced AA7068 Matrix Nanocomposites Produced by Liquid Metallurgy Route. Mater. Sci. Eng. A.

[31] John Joshua K., Vijay S.J., Philip Selvaraj D., Ramkumar P. (2017). Influence of MgO Particles on Microstructural and Mechanical Behaviour of AA7068 Metal Matrix Composites. IOP Conf. Ser. Mater. Sci. Eng..

[32] John Joshua K., Vijay S.J., Philip Selvaraj D. (2018). Effect of Nano TiO_2_ Particles on Microhardness and Microstructural Behavior of AA7068 Metal Matrix Composites. Ceram. Int..

[33] John Joshua K., Vijay S.J., Ramkumar P., Mohanasundaram S. (2020). Effect of ZnO Particles on Microstructure, Microhardness and Wear Behaviour of AA7068 Metal Matrix Composites Synthesized by Powder Metallurgy. Mater. Sci. Forum.

[34] John Joshua K., Ramkumar P., Vijay S.J., Mohanasundaram S., Vijayan S., Subramanian N., Sankaranarayanasamy K. (2021). Microhardness and Microstructural Behavior of AA7068/SiC Metal Matrix Composites Synthesized by Powder Metallurgy. Trends in Manufacturing and Engineering Management.

[35] John Joshua K., Vijay S.J., Ramkumar P., Philip Selvaraj D., Vijayan S., Subramanian N., Sankaranarayanasamy K. (2021). Influence of Graphite Particles on Microhardness and Microstructural Behavior of AA7068 Metal Matrix Composites Processed by Powder Metallurgy. Trends in Manufacturing and Engineering Management.

[36] Nallusamy M., Nandhakumar S., Suriyaprakash M. (2022). Experimental Investigation of Tribological Characteristics on AA7068–ZrB_2_ In-Situ AMCs. Surf. Rev. Lett..

[37] Naresh P., Hussain S.A., Prasad B.D. (2020). Analysis of Dry Sliding Wear Behaviour of AA-7068/TiC MMCs. Int. J. Mater. Eng. Innov..

[38] Singh A.K., Soni S., Rana R.S. (2022). Wear Mechanism Maps for Stir-Squeeze Cast AA7068 Alloy/ZrO_2p_ Composite in Accordance with Normal Load versus Sliding Speed Diagram. Trans. Indian Inst. Met..

[39] Singh A.K., Soni S., Rana R.S. (2023). Mechanical and Sliding Wear Behavior of Stir-Squeeze Cast and T6 Heat-Treated AA7068-ZrO_2p_ Composite. Compos. Interfaces.

[40] Singh A.K., Soni S., Rana R.S., Kumar A., Singh R.K., Chandra G., Srivastava S.K. (2023). Tribological Behavior of High-Strength AA7068 Alloy: Effect of Artificial Aging Temperature. NanoWorld J..

[41] Akinwande A.A., Kumar M.S., Adesina O.S., Adediran A.A., Romanovski V., Salah B. (2023). Tribological Performance of a Novel 7068-Aluminium/Lightweight-High-Entropy-Alloy Fabricated via Powder Metallurgy. Mater. Chem. Phys..

[42] Nambirajan N., Selvakumar N. (2023). Hybrid Composites Reinforced with Graphene and Titanium Carbide in AA7068 Matrix: Evaluation of Mechanical and Tribological Property. Mater. Tehnol..

[43] Ogunbiyi O., Jamiru T., Akinwande A.A. (2025). Mechanical and Wear Characteristics of Aluminium-7068-Composites Structurally Modified with Medium-Entropy-Alloy. Mater. Today Commun..

[44] Ogunbiyi O., Jamiru T., Akinwande A.A., Oketola A. (2025). Physio-Mechanical and Tribological Characterisation of Sintered Aluminium 7068 Modified with NiTiFeAlCu High-Entropy-Alloy for Engineering Applications. Adv. Mater. Process. Technol..

[45] Reddy R.M., Sharma A., Ravi S., Mohanavel V., Raja T., Soudagar M.E.M., Kumar S.S., Obaid S.A., Alahmadi T.A. (2026). Examinations on Hybrid AA7068 Composites Synthesized through Stir Casting Route. J. Mech. Sci. Technol..

[46] Mattli M.R., Shakoor R.A., Matli P.R., Amer Mohamed A.M. (2019). Microstructure and Compressive Behavior of Al–Y_2_O_3_ Nanocomposites Prepared by Microwave-Assisted Mechanical Alloying. Metals.

[47] Qin T., Li G., Wang H., Su W., Dong C., Yu J. (2022). Microstructure and Properties of Microwave-Sintered Nd_2_Fe_14_B_p_/2024 Aluminum-Alloy–Co Composites. Crystals.

[48] Qin T., Fan B., Yu J., Bu C., Zhang J. (2024). Effect of Erbium Micro-Additions on Microstructures and Properties of 2024 Aluminum Alloy Prepared by Microwave Sintering. Crystals.

[49] Dwivedi S.P. (2023). Effect of Microwave Sintering on the Microstructure and Mechanical Properties of Al-Al_2_O_3_-Si_3_N_4_ Hybrid Composite Fabricated by Powder Metallurgy Techniques. Proc. Inst. Mech. Eng. Part C J. Mech. Eng. Sci..

[50] Manoj M., Krishnan G., Mugendiran V. (2023). Effect of Conventional and Microwave Sintering on the Microstructural and Mechanical Properties of AA7075/SiC/ZrC Hybrid MMCs through Powder Metallurgy Route. Matéria (Rio J.).

[51] Mattli M.R., Matli P.R., Khan A., Abdelatty R.H., Yusuf M., Ashraf A.A., Kotalo R.G., Shakoor R.A. (2021). Study of Microstructural and Mechanical Properties of Al/SiC/TiO_2_ Hybrid Nanocomposites Developed by Microwave Sintering. Crystals.

[52] Venkatesh V.S.S., Rao R.N. (2023). Influence of Microwave Sintering Temperatures on Mechanical and Microstructural Behavior of Al/SiC/Snail Shell Hybrid Composite Synthesized Through Powder Metallurgy Technique. Proc. Inst. Mech. Eng. Part C J. Mech. Eng. Sci..

[53] Sutrisna S., Purnomo M.J., Wartono W., Prasetiyo A.B., Riadji M.I.S., Mingwaro W.P. (2026). Optimisation of Microstructure and Wear Properties of Porous Fe-Cu Bearing Materials. Tribol. Mater..

[54] (2023). Standard Test Methods for Density of Compacted or Sintered Powder Metallurgy (PM) Products Using Archimedes’ Principle.

[55] Degen T., Sadki M., Bron E., König U., Nénert G. (2014). The HighScore Suite. Powder Diffr..

[56] Zagorac D., Müller H., Ruehl S., Zagorac J., Rehme S. (2019). Recent Developments in the Inorganic Crystal Structure Database: Theoretical Crystal Structure Data and Related Features. J. Appl. Crystallogr..

[57] (2025). Standard Test Method for Microindentation Hardness of Powder Metallurgy (PM) Materials.

[58] (2025). Standard Test Methods of Compression Testing of Metallic Materials at Room Temperature.

[59] (2023). Standard Test Method for Wear and Friction Testing with a Pin-on-Disk or Ball-on-Disk Apparatus.

[60] Suryanarayana C. (2001). Mechanical Alloying and Milling. Prog. Mater. Sci..

[61] Balcı Ö., Ağaoğulları D., Gökçe H., Duman İ., Öveçoğlu M.L. (2014). Influence of TiB_2_ Particle Size on the Microstructure and Properties of Al Matrix Composites Prepared via Mechanical Alloying and Pressureless Sintering. J. Alloys Compd..

[62] Boschetto A., Giordano V. (2012). Powder Sampling and Characterization by Digital Image Analysis. Measurement.

[63] Gökçe H., Öveçoğlu M.L. (2023). Microstructure-Property Evolution of Mechanically Alloyed Al-20 Wt% Si Matrix Powders and Sintered Composites Reinforced with TiB_2_ Particulates. Eng. Sci. Technol. Int. J..

[64] Özer E., Ayvaz M. (2024). Dry Tribological Behaviour of Microwave-Assisted Sintered AA2024 Matrix Hybrid Composites Reinforced by TiC/B_4_C/Nano-Graphite Particles. Mater. Test..

[65] Ogbonna V.E., Popoola P., Popoola O. (2024). Mechanical and Tribological Properties of Nanoceramic Reinforced Aluminium-Based Nanocomposites for Engineering Applications, Challenges and Recommendations for Future Improvement: A Review. J. Compos. Mater..

[66] Dudina D.V., Georgarakis K., Olevsky E.A. (2023). Progress in Aluminium and Magnesium Matrix Composites Obtained by Spark Plasma, Microwave and Induction Sintering. Int. Mater. Rev..

[67] Newbury D.E., Ritchie N.W.M. (2013). Is Scanning Electron Microscopy/Energy Dispersive X-Ray Spectrometry (SEM/EDS) Quantitative?. Scanning.

[68] Maniammal K., Madhu G., Biju V. (2017). X-Ray Diffraction Line Profile Analysis of Nanostructured Nickel Oxide: Shape Factor and Convolution of Crystallite Size and Microstrain Contributions. Phys. E Low-Dimens. Syst. Nanostruct..

[69] Scardi P., Ermrich M., Fitch A., Huang E.-W., Jardin R., Kuzel R., Leineweber A., Mendoza Cuevas A., Misture S.T., Rebuffi L. (2018). Size–Strain Separation in Diffraction Line Profile Analysis. J. Appl. Crystallogr..

[70] Scherrer P. (1918). Bestimmung Der Größe Und Der Inneren Struktur von Kolloidteilchen Mittels Röntgenstrahlen. Nachrichten Ges. Wiss. Gött. Math. Phys. Kl..

[71] Williamson G.K., Hall W.H. (1953). X-Ray Line Broadening from Filed Aluminium and Wolfram. Acta Metall..

[72] Bragg W.L. (1913). The Diffraction of Short Electromagnetic Waves by a Crystal. Proc. Camb. Philos. Soc..

[73] Henkel L., Koch D., Grathwohl G. (2009). MgAl_2_O_4_-spinel Synthesized by High-energy Ball Milling and Reaction Sintering. J. Am. Ceram. Soc..

[74] Ye G., Troczynski T. (2005). Mechanical Activation of Heterogeneous Sol–Gel Precursors for Synthesis of MgAl_2_O_4_ Spinel. J. Am. Ceram. Soc..

[75] Fabián M., Bottke P., Girman V., Düvel A., Da Silva K.L., Wilkening M., Hahn H., Heitjans P., Šepelák V. (2015). A Simple and Straightforward Mechanochemical Synthesis of the Far-from-Equilibrium Zinc Aluminate, ZnAl_2_O_4_, and Its Response to Thermal Treatment. RSC Adv..

[76] Hedayati A., Golestan Z., Ranjbar K., Borhani G.H. (2011). Effect of Ball Milling on Formation of ZnAl_2_O_4_ by Reduction Reaction of ZnO and Al Powder Mixture. Powder Metall. Met. Ceram..

[77] Chassagne J., Petit C., Meunier C., Valdivieso F. (2022). Preparation of Magnesium and Zinc Aluminate Spinels by Microwave Heating: Influence of the Oxide Precursors on the Phase Composition. Mater. Today Commun..

[78] Spiridigliozzi H., Rouleau O., Kanaev A., Schoenstein F. (2023). Synthesis of MAl_2_O_4_ (M = Mg, Co, Ni, Zn, Ba) Nanopowders by Liquid Impregnation of Nanofibrous Alumina. Ind. Eng. Chem. Res..

[79] Saheb N., Khan M.S. (2018). Compressive Strength and Thermal Properties of Spark Plasma Sintered Al-Al_2_O_3_ Nanocomposite. Sci. Sinter..

[80] Zeng X., Liu W., Xu B., Shu G., Li Q. (2018). Microstructure and Mechanical Properties of Al–SiC Nanocomposites Synthesized by Surface-Modified Aluminium Powder. Metals.

[81] Madhusudhan M., Naveen G.J., Mahesha K. (2017). Mechanical Characterization of AA7068-ZrO_2_ Reinforced Metal Matrix Composites. Mater. Today Proc..

[82] Soni A., Mandloi R.K. (2020). Evaluation of Mechanical Characteristics of Hybrid AA7068/TiB_2_/FA Metal Matrix Composite. Int. J. Mech. Prod. Eng. Res. Dev..

[83] Kolappan S., Arunkumar T., Mohanavel V., Subramani K., Kailasanathan C., Kumaran P., Subbiah R., Suresh Kumar S. (2022). Experimental Investigation on Stir Casted Hybrid Composite AA7068 with SiC and Coconut Shell Fly Ash. Mater. Today Proc..

[84] Singh A.K., Soni S., Rana R.S. (2022). Microstructure Evolution, Mechanical Behavior, and Fracture Analysis of Ultrasonic-Assisted Stir-Squeeze Cast High Strength AA7068/ZrO_2p_/Gr_p_ Composite Under Thermal Aging. Part. Sci. Technol..

[85] Sudheer K.S.D., Srinivasa Rao P., Ratnam C. (2022). Experimental Investigation on Mechanical Properties of AA7068/Marble Dust/Fly Ash Hybrid Composite Processed by Stir Casting Technique. Eng. Res. Express.

[86] Kumar A., Rana R.S., Purohit R. (2022). Microstructure Evolution, Mechanical Properties, and Fractography of AA7068/Si_3_N_4_ Nanocomposite Fabricated Thorough Ultrasonic-Assisted Stir Casting Advanced with Bottom Pouring Technique. Mater. Res. Express.

[87] Zivic F., Babic M., Mitrovic S., Vencl A. (2011). Continuous Control as Alternative Route for Wear Monitoring by Measuring Penetration Depth during Linear Reciprocating Sliding of Ti6Al4V Alloy. J. Alloys Compd..

[88] Archard J.F. (1953). Contact and Rubbing of Flat Surfaces. J. Appl. Phys..

[89] Kumar A., Chaudhari G.P., Nath S.K. (2022). Correlation of Microstructure with Corrosion Performance in High Zinc 7068 Aluminum Alloy Aged Using Different T6 Conditions. Mater. Charact..

